# Tracking longitudinal biomarkers in burn patients with sepsis and acute kidney injury: an unsupervised clustering approach

**DOI:** 10.1186/s40001-023-01268-3

**Published:** 2023-08-25

**Authors:** Myongjin Kim, Dohern Kym, Jun Hur, Jongsoo Park, Jaechul Yoon, Yong Suk Cho, Wook Chun, Dogeon Yoon

**Affiliations:** 1 Department of Surgery and Critical Care, Burn Center, Hangang Sacred Heart Hospital, College of Medicine, Hallym University Medical Center, 12, Beodeunaru-Ro 7-gil, Youngdeungpo-gu, Seoul, 07247 Korea; 2 Burn Institutes, Hangang Sacred Heart Hospital, Hallym University Medical Center, 12, Beodeunaru-Ro 7-gil, Youngdeungpo-gu, Seoul, 07247 Korea

**Keywords:** Longitudinal, Unsupervised, AKI, Sepsis, Burns

## Abstract

**Background:**

Sepsis is a grave medical disorder characterized by a systemic inflammatory response to infection. Furthermore, it is a leading cause of morbidity and mortality, especially in hospitalized patients. Acute kidney injury (AKI) is a common complication of sepsis and is associated with increased morbidity and mortality. Patients with burns are particularly vulnerable to developing sepsis and AKI due to the extensive tissue damage and immune suppression resulting from burn injury. In this study, unsupervised clustering algorithms were used to track longitudinal biomarkers in patients with burns and assess their impact on mortality.

**Methods:**

This retrospective study included adult patients with burns aged ≥ 18 years, who were admitted to the burn intensive care unit of Hallym University and Hangang Sacred Heart Hospital between July 2010 and December 2021. The patients were divided into two subgroups: those with sepsis (538 patients) and those without sepsis (826 patients). The longitudinal biomarkers were grouped into three clusters using the *k*-means clustering algorithm. Each cluster was assigned a letter from A to C according to its mortality rate.

**Results:**

The odds ratio (OR) of pH was 9.992 in the positive group and 31.745 in the negative group in cluster C. The OR for lactate dehydrogenase (LD) was 3.704 in the positive group and 6.631 in the negative group in cluster C. The OR for creatinine was 2.784 in the positive group and 8.796 in the negative group in cluster C. The OR for blood urea nitrogen (BUN) in the negative group was 0.348, indicating a negative predictor of mortality. Regarding the application of Continuous Renal Replacement Therapy (CRRT) and ventilation, ventilation was significant in both groups. In contrast, CRRT application was not significant in the sepsis-positive group. Furthermore, it was not selected as a variable in the negative group.

**Conclusions:**

The pH, LD, and creatinine were significant in both groups, while lactate and platelets were significant in the sepsis-positive group. In addition, albumin, glucose, and BUN were significant in the sepsis-negative group. Continuous renal replacement therapy was not significant in either group. However, the use of a ventilator was associated with poor prognosis.

**Supplementary Information:**

The online version contains supplementary material available at 10.1186/s40001-023-01268-3.

## Background

Sepsis is a serious medical condition characterized by a systemic inflammatory response to infection. It is a leading cause of morbidity and mortality, particularly in hospitalized patients [1]. Acute kidney injury (AKI) is a common complication of sepsis associated with increased morbidity and mortality rates. Patients with burns are particularly at risk of developing sepsis and AKI due to extensive tissue damage and immune suppression from burn injury [2, 3]. The pathophysiology of sepsis-related AKI in patients with burns is complex and not fully understood. However, it is thought to be due to a combination of factors, including inflammation, oxidative stress, and microvascular dysfunction in late-onset AKI [4]. The immune system plays a crucial role in developing sepsis and AKI; however, burn injuries can significantly alter the immune function. Moreover, burn injury damages the vasculature and microcirculation, leading to impaired perfusion and oxygen delivery to the kidneys. These effects can result in ischemia and oxidative stress, further damaging the kidneys and contributing to AKI development [5].

The diagnosis of sepsis-related AKI in critically ill patients can be challenging due to other factors that can affect kidney function, such as fluid and electrolyte imbalances, medications, and the effects of the burn injury itself [5]. It is important for clinicians to carefully monitor patients with burns for signs and symptoms of sepsis and AKI, including fever, hypotension, tachycardia, oliguria, and changes in mental status. Routine laboratory tests such as serum creatinine, blood urea nitrogen (BUN), lactate, and urine output can also help diagnose and monitor AKI. Despite advances in diagnosing and managing sepsis and AKI, the outcomes of patients with burns with these conditions remain poor [2, 6].

Time-event data or time-series data, are an important area of focus in biostatistics and its application in medical research. Unsupervised clustering algorithms can identify groups of patients with similar clinical characteristics in heterogeneous medical conditions, providing insight into the mechanisms of disease development and underlying heterogeneity [7–9]. Using clustering algorithms and tracking longitudinal biomarkers, we can better understand the progression of AKI and sepsis and their impact on mortality in patients with burns. Moreover, we will also gain insight into the underlying mechanisms of disease development and heterogeneity in these patients. Unsupervised clustering algorithms are especially important for distinguishing and analyzing patient groups without being affected by the heterogeneity of diseases, such as severe burn injuries [10]. In doing so, we can uncover clues to the underlying mechanisms of these hidden diseases.

In this study, we aimed to use these algorithms to distinguish differences in the longitudinal changes in routine biomarkers, understand their clinical significance in patients with burns, use unsupervised clustering algorithms to track longitudinal biomarkers in patients with burns, and assess their impact on mortality.

## Methods

### Study site and patients

The present study adopted a retrospective design, comprising adult patients with burns aged ≥ 18 years, who were admitted to the burn intensive care unit (BICU) of Hallym University and Hangang Sacred Heart Hospital between July 2010 and December 2021. AKI was diagnosed using the Acute Kidney Injury Network criteria (Stage 1: creatinine increased by 1.5 times the baseline value or an absolute increase in creatinine of at least 0.3 mg/dL within 48 h, or urine production of less than 0.5 mL/kg for 6 h; Stage 2: creatinine increased by two times the baseline value or urine production of less than 0.5 mL/kg for 12 h; Stage 3: creatinine increased by thrice the baseline value or greater than 4 mg/dL or an absolute increase in creatinine of at least 0.3 mg/dL within 48 h, urine output of less than 0.3 mL/kg for 24 h or requiring continuous renal replacement therapy (CRRT) regardless of the status). Baseline creatinine levels were only included if they were measured within 3 months before the burn injury. In cases where the measured results were unavailable, we estimated the baseline creatinine levels using the modification of diet in the renal disease equation [11]. Sepsis was diagnosed using the Sequential Organ Failure Assessment (SOFA) score, with a score of ≥ 2 according to the sepsis-3 criteria indicating the presence of an acute abnormal change [1]. Patients diagnosed with AKI were divided into two groups: sepsis-positive AKI and sepsis-negative AKI, based on the diagnosis of sepsis within 1 week of AKI diagnosis. All patients in both groups underwent routine laboratory tests, including complete blood counts, arterial blood gas analyses, electrolyte levels, and routine chemistry, at least every 3–4 days during their intensive care unit (ICU) stay.

### Data collection and missing value

The clinical database warehouse (CDW) of Hallym University Medical Center provided a comprehensive set of clinical longitudinal data collected prospectively. All relevant variables were collected from the point of admission until the time of death in the nonsurvival group and until discharge from the BICU in the survival group. The worst biomarker values were obtained when measurements were taken multiple times daily. Demographic characteristics, such as age, sex, and extent of burns were determined using modified Lund and Browder charts. Other factors included the type of burn, length of stay in the BICU, presence of inhalation injury, and routine laboratory test results from the CDW. The primary outcome was the in-hospital mortality rate within 60 days. Upon admission, injury severity was assessed using the Abbreviated Burn Severity Index (ABSI) and revised Baux (rBaux) index, as well as our center's newly developed Hangang score [12]. Additionally, Acute Physiology and Chronic Health Evaluation Score (APACHE) IV and SOFA scores were calculated daily using routine laboratory results. Missing values for these longitudinal variables were imputed using the copyMean method, a commonly used method for predicting missing data in longitudinal studies [13]. This method involves interpolating the values immediately surrounding the missing data using a straight line and imputing the missing values using the last observation carried forward, or the following observation carried backward methods. The missing longitudinal data are shown in Additional file 1: Figure S1. The study was approved by the Institutional Review Board of Hangang Sacred Heart Hospital, and the requirement for informed consent was waived due to the study's retrospective nature and the lack of interventions.

### Statistical analysis

The basic demographic characteristics were as follows: continuous numerical variables with a normal distribution were presented as mean ± standard deviation (SD), while those with a non-normal distribution were presented as the median and interquartile range (IQR). Depending on the normality of the distribution, a paired *t* test or Wilcoxon signed-rank test was used to determine the differences between the two groups. Categorical variables were expressed as percentages and compared between groups using the chi-square test or Fisher's exact test, as appropriate. Longitudinal biomarkers were grouped into three clusters using the *k*-means clustering algorithm, a useful method for clustering longitudinal data based on their shape. Each cluster was assigned a letter from A to C according to its mortality rate using the kmlShape package in the R-project program [14]. Univariate and multivariate logistic regression analyses for mortality were performed, with highly correlated biomarkers (multicollinearity) removed. Furthermore, the stepwise selection was used to select the final set of biomarkers for analysis. All tests were two-tailed, and *P* values less than 0.05 were considered statistically significant. All analyses were performed using the Statistical R-project program version 4.2.2.

## Results

### Baseline characteristics of study population

**Fig. 1:**
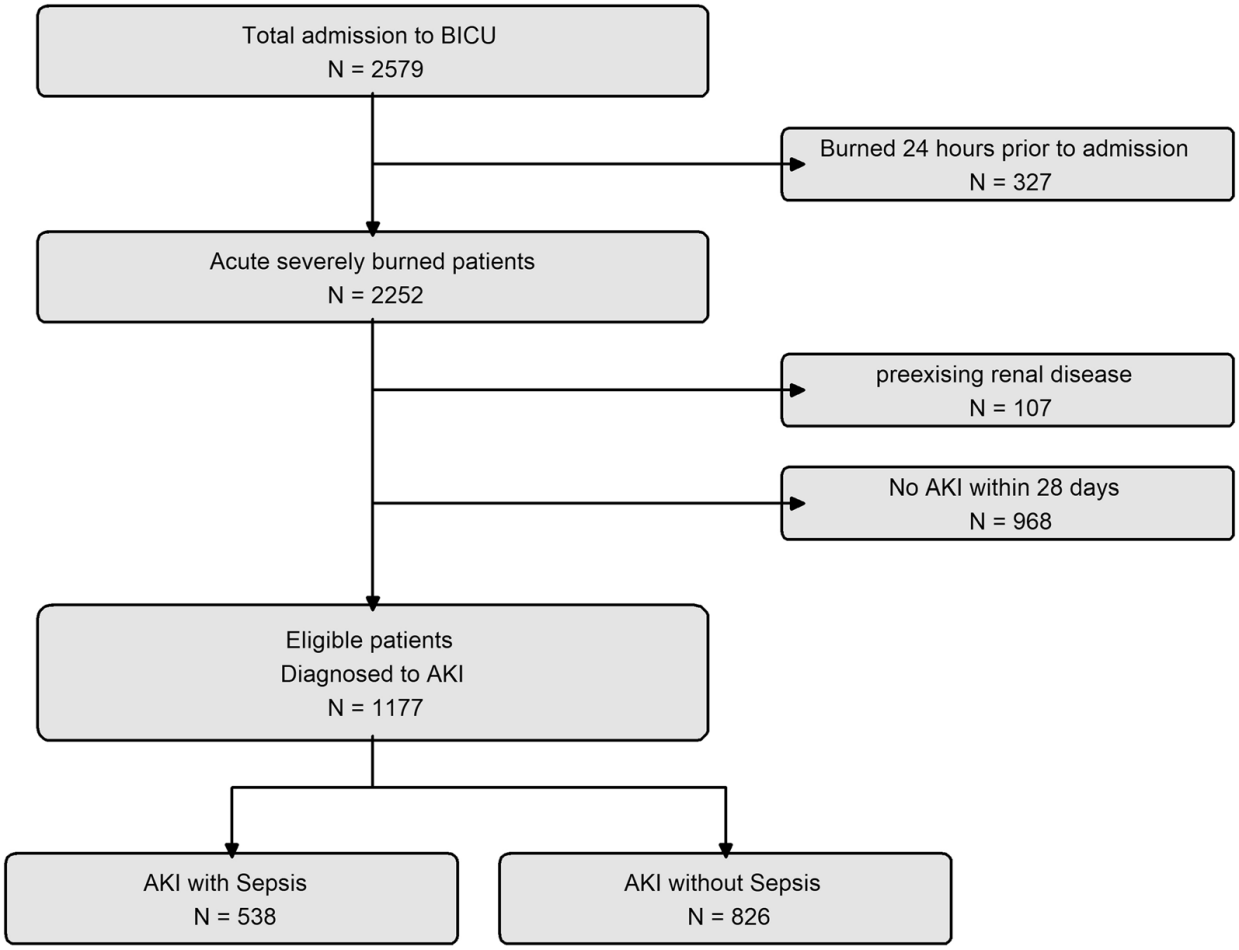
Flowchart for enrolling patients

**Fig. 2:**
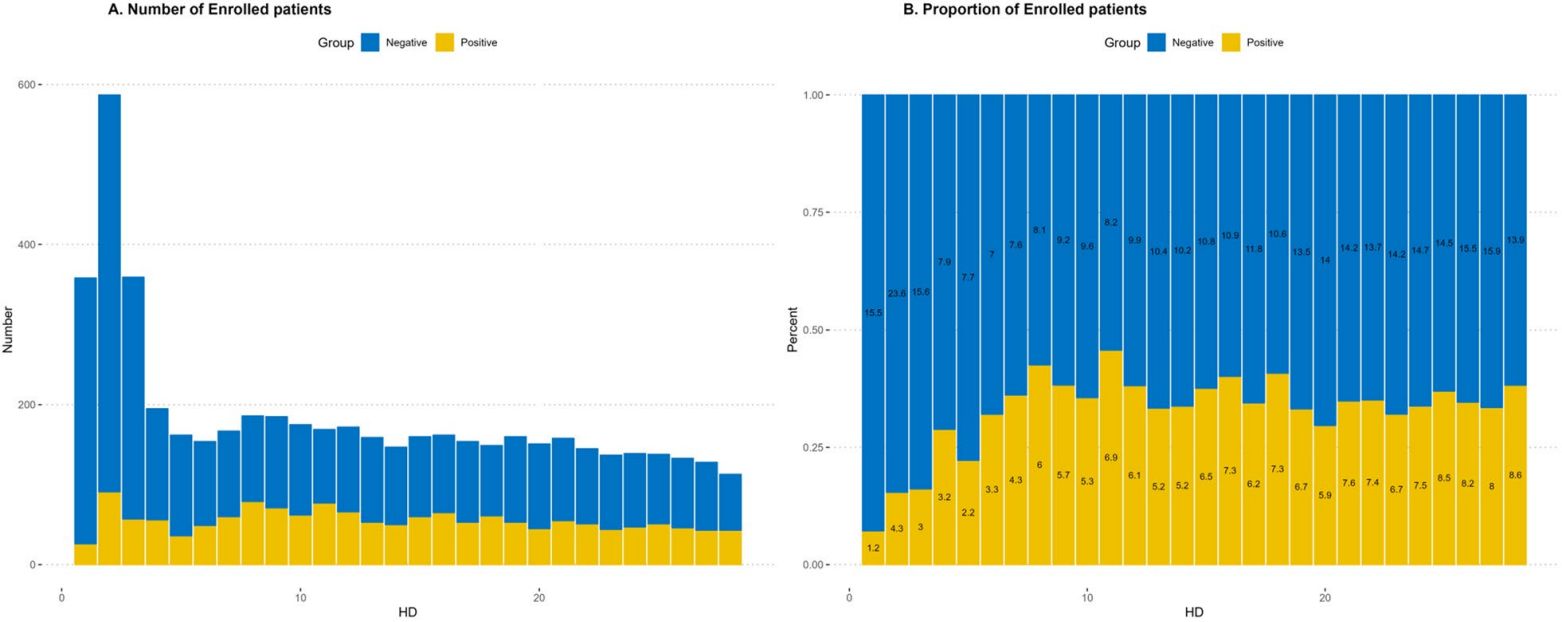
The number and proportion of enrolled patients

In total, 2579 patients were admitted to the BICU at Hallym University Hangang Hospital between July 2010 and December 2021. Of the 2579 patients, we excluded those who had been hospitalized for more than 2 days after their burns. From the remaining 2252 patients, we further excluded 107 patients who had the following conditions: (1) an existing health condition affecting renal function, such as chronic kidney disease, liver disease, cardiac disease, or diabetes mellitus; and (2) a specific medical history in the past. In addition, we excluded 968 patients who did not develop AKI. The remaining 1177 patients were then divided into two groups based on the presence or absence of sepsis within 1 week of their diagnosis, referred to as the sepsis-positive group with 538 patients and the sepsis-negative group with 826 patients, respectively. A total of 187 patients developed both sepsis-positive and sepsis-negative AKI. The study enrollment process is depicted in Fig. 1. Furthermore, the number and proportion of AKI cases according to hospital day were higher in the sepsis-negative group as compared to that in the sepsis-positive group (Fig. 2).

Among the eligible 1177 patients, 478 patients (40.6%) died. The overall median age was 52 years (interquartile range [IQR] 42–63 years), and there was a male predominance (81.1%). The median total body surface area (TBSA) affected by burns was 37% and inhalation injury was present in 572 (48.6%) patients. The median length of stay in the ICU was 17 days (IQR 6–32 days). The median Acute Physiology and Chronic Health Evaluation (APACHE) IV, Sequential Organ Failure Assessment (SOFA), Abbreviated Burn Severity Index (ABSI), rBaux, and Hangang scores were 54, 4, 9, 101, and 139, respectively. The most common comorbidities were hypertension (20.0%), diabetes mellitus (DM) (9.8%), hyperlipidemia (2.7%), and cardiovascular disease (2.9%) (Table 1).

**Table 1:** Demographics for patients with burns in enrolled patients

Group	Variables	Overall, *N* = 1177	Survivors, *N* = 699 (59.4%)	Non_Survivors, *N* = 478 (40.6%)	*p* value
Demographics	Patient age				< 0.001
	Median [IQR]	52 [42, 63]	50 [41, 60]	55 [46, 67]	
	Sex				0.375
	Male	955 (81.1%)	573 (82.0%)	382 (79.9%)	
	Female	222 (18.9%)	126 (18.0%)	96 (20.1%)	
	Type				< 0.001
	FB	908 (77.1%)	485 (69.4%)	423 (88.5%)	
	SB	113 (9.6%)	84 (12.0%)	29 (6.1%)	
	EB	106 (9.0%)	93 (13.3%)	13 (2.7%)	
	ChB	15 (1.3%)	11 (1.6%)	4 (0.8%)	
	CoB	35 (3.0%)	26 (3.7%)	9 (1.9%)	
	TBSA				< 0.001
	Median [IQR]	37 [21, 61]	25 [16, 40]	64 [41, 84]	
	Inhalation	572 (48.6%)	260 (37.2%)	312 (65.3%)	< 0.001
	LOICU				< 0.001
	Median [IQR]	17 [6, 32]	24 [8, 40]	11 [4, 20]	
Severity Scores	ABSI				< 0.001
	Median [IQR]	9 [7, 11]	7 [6, 9]	12 [10, 14]	
	rBaux				< 0.001
	Median [IQR]	101 [79, 127]	86 [70, 101]	131 [110, 150]	
	Hangang				< 0.001
	Median [IQR]	139 [123, 158]	127 [116, 139]	164 [149, 179]	
	APACHE IV				< 0.001
	Median [IQR]	54 [37, 76]	42 [29, 56]	74 [60, 94]	
	SOFA				< 0.001
	Median [IQR]	4 [3, 7]	3 [2, 5]	7 [5, 10]	
Comorbidities	Hypertension	235 (20.0%)	132 (18.9%)	103 (21.5%)	0.262
	Diabetes Mellitus	115 (9.8%)	55 (7.9%)	60 (12.6%)	0.008
	Tuberculosis	17 (1.4%)	10 (1.4%)	7 (1.5%)	0.962
	Hepatobiliary	26 (2.2%)	18 (2.6%)	8 (1.7%)	0.301
	Cardiovascular	34 (2.9%)	23 (3.3%)	11 (2.3%)	0.320
	CVA	17 (1.4%)	13 (1.9%)	4 (0.8%)	0.149
	Cancer	31 (2.6%)	16 (2.3%)	15 (3.1%)	0.372
	Hyperlipidemia	32 (2.7%)	20 (2.9%)	12 (2.5%)	0.716
	Other	298 (25.3%)	189 (27.0%)	109 (22.8%)	0.101

In the sepsis-positive group, 305 patients (56.7%) died. The overall median age was 54 years (IQR 44–64 years), and there was a male predominance (83.5%). The median TBSA affected by burns was 47% and inhalation injury was present in 281 (52.2%) patients. The median length of stay in the ICU was 22 days (IQR 12–37 days). The median APACHE IV, SOFA, ABSI, rBaux, and Hangang scores were 66, 7, 10, 110, and 144, respectively. The most common comorbidities were hypertension (22.3%), DM (10.8%), hyperlipidemia (2.6%), and cardiovascular disease (3.0%) (Table 1). In the sepsis-negative group, 269 patients (32.6%) died. The overall median age was 51 years (IQR 41–62 years), and there was a male predominance (80.4%). The median TBSA affected by burns was 32%, and inhalation injury was present in 388 (47.0%) patients. The use of CRRT and mechanical ventilation was more prevalent in the sepsis-positive group at 42.4% and 88.4%, respectively, compared to that in the sepsis-negative group (Table 2).

**Table 2:** Characteristics in the sepsis-positive/negative groups

Group	Variables	Sepsis positive				Sepsis negative			
		Overall, *N* = 538	Survivors, *N* = 233 (43.3%)	Non_Survivors, *N* = 305 (56.7%)	*p* value	Overall, *N* = 826	Survivors, *N* = 557 (67.4%)	Non_Survivors, *N* = 269 (32.6%)	*p* value
Demographics	Patient Age				0.621				< 0.001
	Median [IQR]	54 [44, 64]	54 [44, 63]	54 [44, 65]		51 [41, 62]	49 [40, 58]	55 [46, 71]	
	Sex				0.899				0.123
	Male	449 (83.5%)	195 (83.7%)	254 (83.3%)		664 (80.4%)	456 (81.9%)	208 (77.3%)	
	Female	89 (16.5%)	38 (16.3%)	51 (16.7%)		162 (19.6%)	101 (18.1%)	61 (22.7%)	
	Type				0.004				< 0.001
	FB	436 (81.0%)	173 (74.2%)	263 (86.2%)		630 (76.3%)	388 (69.7%)	242 (90.0%)	
	SB	52 (9.7%)	30 (12.9%)	22 (7.2%)		74 (9.0%)	60 (10.8%)	14 (5.2%)	
	EB	28 (5.2%)	18 (7.7%)	10 (3.3%)		88 (10.7%)	82 (14.7%)	6 (2.2%)	
	ChB	8 (1.5%)	6 (2.6%)	2 (0.7%)		9 (1.1%)	6 (1.1%)	3 (1.1%)	
	CoB	14 (2.6%)	6 (2.6%)	8 (2.6%)		25 (3.0%)	21 (3.8%)	4 (1.5%)	
	TBSA				< 0.001				< 0.001
	Median [IQR]	47 [28, 67]	32 [20, 48]	61 [40, 80]		32 [20, 60]	25 [16, 37]	73 [46, 88]	
	Inhalation	281 (52.2%)	106 (45.5%)	175 (57.4%)	0.006	388 (47.0%)	196 (35.2%)	192 (71.4%)	< 0.001
	LOICU				< 0.001				< 0.001
	Median [IQR]	22 [12, 37]	37 [23, 60]	15 [9, 24]		14 [5, 31]	21 [7, 36]	7 [2, 18]	
Intervention	CRRT apply	228 (42.4%)	45 (19.3%)	183 (60.0%)	< 0.001	63 (7.6%)	10 (1.8%)	53 (19.7%)	< 0.001
	Ventilator apply	465 (86.4%)	162 (69.5%)	303 (99.3%)	< 0.001	465 (56.3%)	202 (36.3%)	263 (97.8%)	< 0.001
Severity Scores	ABSI				< 0.001				< 0.001
	Median [IQR]	10 [1, 8]	9 [7, 10]	11 [10, 13]		8 [7, 11]	7 [6, 9]	13 [10, 14]	
	rBaux				< 0.001				< 0.001
	Median [IQR]	110 [94, 131]	97 [82, 108]	124 [107, 141]		96 [74, 120]	83 [67, 98]	137 [114, 154]	
	Hangang				< 0.001				< 0.001
	Median [IQR]	144 [132, 161]	132 [123, 141]	157 [144, 170]		135 [120, 155]	126 [115, 136]	169 [153, 183]	
	APACHE IV				< 0.001				< 0.001
	Median [IQR]	66 [49, 86]	53 [40, 70]	77 [60, 93]		49 [32, 71]	40 [28, 53]	78 [62, 101]	
	SOFA				< 0.001				< 0.001
	Median [IQR]	7 [4, 10]	5 [3, 7]	9 [6, 11]		4 [2, 6]	3 [2, 4]	7 [5, 10]	
Comobidities	Hypertension	120 (22.3%)	52 (22.3%)	68 (22.3%)	0.995	155 (18.8%)	99 (17.8%)	56 (20.8%)	0.294
	Diabetes Mellitus	58 (10.8%)	24 (10.3%)	34 (11.1%)	0.754	72 (8.7%)	36 (6.5%)	36 (13.4%)	< 0.001
	Tuberculosis	9 (1.7%)	5 (2.1%)	4 (1.3%)	0.511	10 (1.2%)	5 (0.9%)	5 (1.9%)	0.308
	Hepatobiliary	13 (2.4%)	8 (3.4%)	5 (1.6%)	0.179	19 (2.3%)	15 (2.7%)	4 (1.5%)	0.279
	Cardiovascular	16 (3.0%)	10 (4.3%)	6 (2.0%)	0.116	25 (3.0%)	18 (3.2%)	7 (2.6%)	0.621
	CVA	10 (1.9%)	7 (3.0%)	3 (1.0%)	0.110	11 (1.3%)	9 (1.6%)	2 (0.7%)	0.518
	Cancer	16 (3.0%)	6 (2.6%)	10 (3.3%)	0.634	21 (2.5%)	12 (2.2%)	9 (3.3%)	0.308
	Hyperlipidemia	14 (2.6%)	7 (3.0%)	7 (2.3%)	0.609	22 (2.7%)	15 (2.7%)	7 (2.6%)	0.939
	Other	127 (23.6%)	65 (27.9%)	62 (20.3%)	0.041	212 (25.7%)	147 (26.4%)	65 (24.2%)	0.492

### Predictors in both groups

**Fig. 3:**
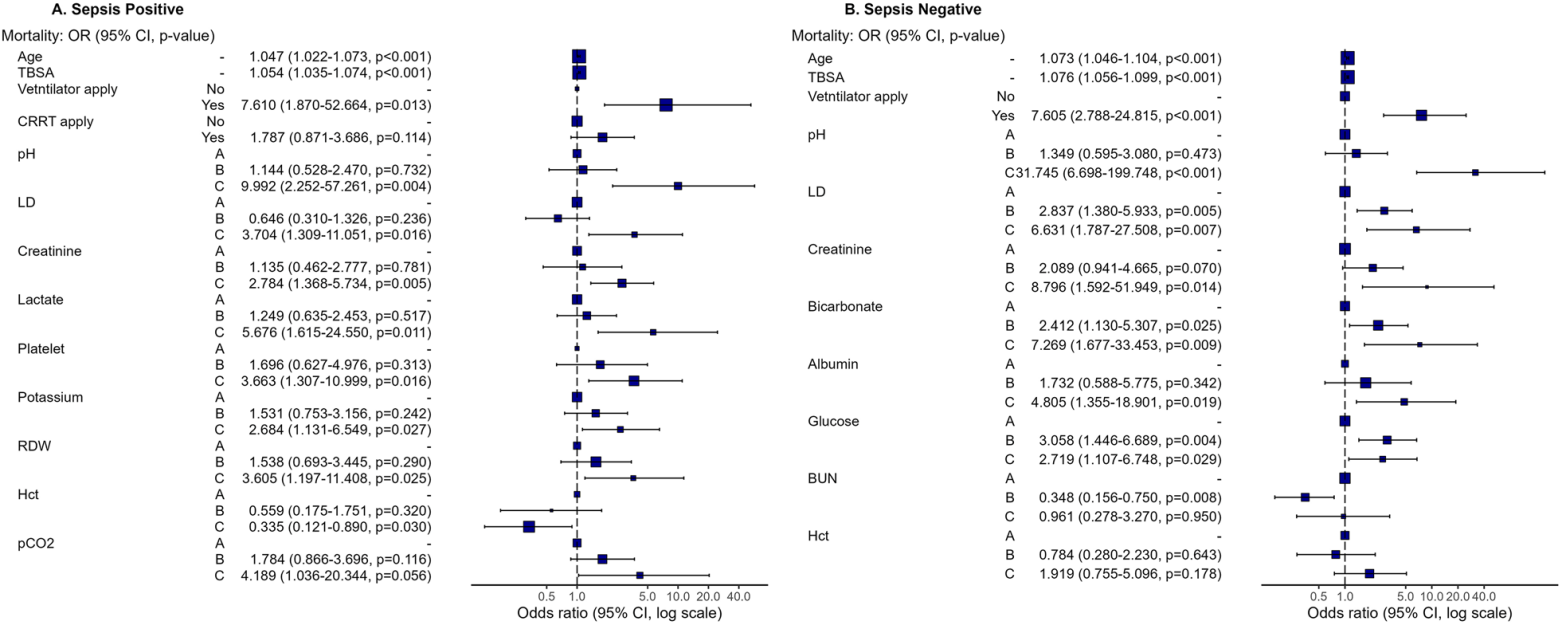
Odds ratio of multiple logistic regression for mortality in both groups

In this study, we evaluated a total of 23 biomarkers that were checked at least every 4 days. In the sepsis-positive group, among these biomarkers, pH, LD, creatinine, lactate, and platelet were statistically significant. In the sepsis-negative group, pH, LD, creatinine, bicarbonate, albumin, glucose, and BUN were statistically significant. A subset of three biomarkers—pH, LD, and creatinine—exhibited statistical significance in both groups. The odds ratio (OR) of pH was 9.992 in cluster C in the positive group and 31.745 in cluster C in the negative group. The OR of LD was 3.704 in cluster C in the positive group and 6.631 in cluster C in the negative group. The OR of creatinine was 2.784 in cluster C in the positive group and 8.796 in cluster C in the negative group. The OR of BUN in the negative group was 0.348, which is below 1, indicating an inverse prediction for mortality. In terms of the application of CRRT and ventilation, only ventilation was significant in both groups. CRRT application was not significant in the sepsis-positive group, and was not even selected as a variable in the sepsis-negative group (Fig. 3).

### Predictors, longitudinal profile clusters, and mortality: a Sankey diagram approach

A longitudinal assessment of nine salient biomarkers—pH, LD, creatinine, lactate, platelets, bicarbonate, albumin, glucose, and BUN—was carried out utilizing k-means clustering facilitated by the kmlShape package. The ensuing longitudinal profiles are illustrated in Additional file 1: Figures S2–S10. To further scrutinize the interconnections among predictors, clusters, and mortality, an interactive Sankey diagram was constructed and can be accessed via the project website [15]. This visualization instrument enables a comprehensive exploration of the associations between the aforementioned variables within the study's framework.

In pH, the longitudinal profiles showed a slightly declining trend from day 1 (7.17) to day 7 (7.14) in cluster C, which had the highest mortality rate (98.1%) in the sepsis-positive group. In the sepsis-negative group, the pH level slightly increased from 7.38 to 7.42 on day 7 in cluster A, which had the lowest mortality rate (6.0%) (Additional file 1: Figure S11 and Table S1). In LD, the longitudinal profiles showed the highest levels in cluster C in both groups. The median levels in cluster C were 592 in the sepsis-positive group and 593 in the sepsis-negative group (Additional file 1: Figure S12 and Table S2). In creatinine, the longitudinal profiles showed a similar pattern in both groups; however, cluster C was in the middle of the plot in the sepsis-positive group and was in the upper part of the plot in the sepsis-negative group (Additional file 1: Figure S13 and Table S3) This suggests that clusters with lower creatinine levels had a worse prognosis. In the sepsis-positive group, the median platelet level ranged from 322 on day 1 to 391 on day 7. In the sepsis-negative group, the median pH level ranged from 216 on day 1 to 250 on day 7. The mean median level in cluster A was 189, which was lower than that of cluster B (415) (Additional file 1: Figure S14 and Table S4). The raincloud plots, characteristics, and levels of the other biomarkers under investigation are depicted in Additional file 1: Figures S15–S19 and detailed in Additional file 1: Tables S5–S9.

## Discussion

### Key findings

This study evaluated 23 biomarkers in the sepsis-positive and sepsis-negative groups to determine their significance in predicting mortality. The pH, LD, and creatinine levels were significant in both groups. Lactate and platelet counts were significant in the positive group, while albumin, glucose, and BUN were significant in the sepsis-negative group. Continuous Renal Replacement Therapy was not significantly different between the groups. However, ventilator use was associated with a poor prognosis. This study also analyzed the longitudinal profiles of nine biomarkers and found that clusters with lower creatinine levels had worse prognoses in the sepsis-positive group. We inferred that this was because CRRT was used more frequently in the sepsis-positive group, resulting in relatively lower creatinine levels.

### Relationship to previous studies

Sepsis is the leading cause of AKI in the ICU, and 15–20% of patients with sepsis-associated AKI (SA-AKI) require renal replacement therapy [16]. Sepsis accounts for > 50% of AKI cases, with a mortality rate as high as 40% [17]. Other risk factors are often present when AKI occurs in sepsis [18]. In our study, although the application of CRRT was not a significant factor, CRRT was more frequently applied in the sepsis-positive group because CRRT could be applied to patients with electrolyte imbalances or severe acidosis, induced by not only AKI but also sepsis [19]. Despite its significance, the treatment recommendations for SA-AKI [20] are not clearly defined, highlighting the need for timely and effective monitoring using longitudinal biomarkers, such as pH, LD, and creatinine, as we found in this study, as well as treatment of the underlying infection, prevention of secondary kidney injury, and optimization of systemic hemodynamics.

Evidence suggests that the pH of the body's fluids, specifically arterial blood pH, plays a role in the development and severity of AKI in critically ill patients [21]. Patients with burns with sepsis with a lower arterial blood pH are more likely to develop AKI and have a higher mortality rate than those with a higher pH [22]. This association may be because a lower pH is associated with decreased perfusion and oxygen delivery to the kidneys, leading to tissue damage and impaired function [17]. The pH level is a measure of the acid–base balance in the body. It is typically associated with lactate and bicarbonate, which both play a role in the acid–base balance. This study observed a statistically significant relationship between pH and lactate and bicarbonate levels. LD is an enzyme found in many tissues, including kidneys. Elevated levels of LD in the blood have been linked to AKI, particularly sepsis [23]. LD is thought to play a role in the development of AKI through its effects on the micro-vasculature, leading to ischemia and tissue damage [24]. Creatinine is a waste product produced by the breakdown of muscle tissue and is filtered out by the kidneys. Elevated creatinine levels in the blood can be a sign of AKI, which was also observed in patients with burns with sepsis-positive AKI in this study.

Lactate, a byproduct of anaerobic metabolism, is often elevated in conditions of severe sepsis or septic shock. This is due to the body's cells switching to a less efficient form of energy production that doesn't require oxygen when they are not receiving enough of it. High levels of lactate in the blood can therefore be a sign of severe sepsis or septic shock, and lactate clearance (the reduction in lactate levels over time) is often used as a marker of the body's response to treatment for sepsis [25, 26]. Platelets, also known as thrombocytes, are small disk-shaped blood cells involved in clotting. During sepsis, the body's immune system responds to infection or injury by releasing substances, such as cytokines and chemokines, which can trigger inflammation. This inflammatory response can lead to the activation and aggregation of platelets, which can contribute to the development of blood clots. These clots can occlude blood vessels, leading to impaired blood flow and tissue damage [27]. In our study, the platelet count and lactate levels were significantly higher in the sepsis-positive group.

In our study, we observed that albumin, glucose, and BUN were significant markers in the sepsis-negative group. Albumin, a major protein in the blood, plays a pivotal role in maintaining oncotic pressure and facilitating the transport of various substances throughout the body. Reduced serum albumin levels could be indicative of malnutrition, liver disease, inflammation, or increased capillary permeability, conditions that are often present in critically ill patients [28]. Glucose levels, on the other hand, can be influenced by a variety of factors, including stress, illness, and certain medications. Both hyperglycemia and hypoglycemia have been associated with poorer outcomes in critically ill patients, underscoring the importance of maintaining optimal glucose levels. BUN is produced in the liver and excreted by the kidneys. Elevated BUN levels may indicate AKI because the kidneys remove excess urea from the blood. BUN levels may also be elevated in cases of dehydration, malnutrition, or liver disease and have been reported as predictors of AKI in patients with burns [29]. In this study, albumin and glucose were significant markers, although they are typically unknown predictors of AKI. However, low serum albumin levels have been associated with an increased risk of death in patients with severe sepsis [30]. Additionally, elevated glucose levels have been linked to an increased risk of complications in patients with burns, including infection and delayed wound healing [31].

### Strengths and limitations

Our study presents several notable strengths. Firstly, the innovative use of longitudinal data and k-means clustering algorithms distinguishes our approach. Secondly, we employed deep learning mechanisms, which allowed us to leverage artificial intelligence in analyzing a comprehensive dataset. This facilitated the identification of significant biomarkers in AKI with and without sepsis in patients with burns. The use of deep learning not only augmented the efficiency and accuracy of our analysis but also mitigated the potential for human bias in biomarker selection. This unbiased approach is particularly salient in medical research, where the identification of reliable and significant biomarkers can substantially enhance diagnostic and prognostic accuracy. Furthermore, the longitudinal nature of our data offered a dynamic view of patient progression, enabling us to monitor changes in biomarker levels over time and gain a deeper understanding of their role in patient outcomes. This temporal perspective, often absent in traditional cross-sectional studies, adds a valuable dimension to our research.

However, our study is not without limitations. Conducting our study at a single center potentially introduces geographical bias, as the patient population at our center may possess unique characteristics unrepresentative of the broader population. This could affect the generalizability of our findings and limit their applicability to other settings or populations. Additionally, while our dataset is comprehensive, it is subject to the limitations inherent in retrospective studies, including potential inaccuracies in medical records and the inability to control for all confounding variables.

Despite these limitations, our study provides valuable insights into the role of biomarkers in AKI with and without sepsis in patients with burns. Future research should aim to validate our findings in multicenter studies and prospective designs, which could help to overcome these limitations and further enhance our understanding of this complex issue.

## Conclusions

The pH, LD, and creatinine were found to be significant in both groups, while lactate and platelets were significant in the sepsis-positive group. Furthermore, albumin, glucose, and BUN were significant in the sepsis-negative group. CRRT was not significant in either group. In addition, the use of a ventilator was associated with poor prognosis. However, the association between these markers and sepsis-associated AKI in patients with burns is complex. It requires further research to fully understand the mechanisms underlying this association and develop effective strategies for preventing and managing AKI in this patient population.

### Supplementary Information

**Additional file 1:** Supplementary material.

## Data Availability

The datasets used and/or analyzed during the current study are available from the corresponding author on reasonable request.

## Abbreviations

AKI: Acute Kidney Injury
BUN: Blood Urea Nitrogen
CRRT: Continuous Renal Replacement Therapy
ICU: Intensive Care Unit
SD: Standard Deviation
IQR: Interquartile Range
SOFA: Sequential Organ Failure Assessment
APACHE: Acute Physiology and Chronic Health Evaluation
ABSI: Abbreviated Burn Severity Index
rBaux: Revised Baux Index
CDW: Clinical Database Warehouse
BICU: Burn Intensive Care Unit
TBSA: Total Body Surface Area
LD: Lactate Dehydrogenase
