# Supplementary material for: Tracking longitudinal biomarkers in burn patients with sepsis and acute kidney injury: an unsupervised clustering approach
Source: Eur J Med Res. 2023 Aug 25;28:295. doi: 10.1186/s40001-023-01268-3 (PMC10464319; doi:10.1186/s40001-023-01268-3)
Supplement: Supplementary file 1 — Additional file 1: Supplementary material. [file 40001_2023_1268_MOESM1_ESM.docx]

**Additional material**

**Tracking Longitudinal Biomarkers in Burn Patients with Sepsis and Acute Kidney Injury: An Unsupervised Clustering Approach**

Myongjin Kim1, Dohern Kym1,2*+, Jongsoo Park1, Jun Hur1+ , Jaechul Yoon1, Yong-Suk Cho1, Wook Chun1,2, Dogeon Yoon2

1Department of Surgery and Critical Care, Burn Center, Hangang Sacred Heart Hospital, Hallym University Medical Center, 12, Beodeunaru-ro 7-gil, Youngdeungpo-gu, Seoul, Korea, 07247

2Burn Institutes, Hangang Sacred Heart Hospital, Hallym University Medical Center, 12, Beodeunaru-ro 7-gil, Youngdeungpo-gu, Seoul, Korea, 07247

+Dohern Kym and Jun Hur contributed equally in this work as corresponding author.

*Corresponding authors

Department of Surgery and Critical Care, Burn Center, Hangang Sacred Heart Hospital, College of Medicine, Hallym University 12, Beodeunaru-ro 7-gil, Youngdeungpo-gu, Seoul, Korea, 07247

Tel. 82-2-2639-5446, Fax. 82-2-2678-4386, E-mail: dohern@hallym.or.kr

Contents

[STROBE Statement—Checklist of items that should be included in reports of cohort studies 4](#_Toc133519624)

[Figure S1. Plot of Missingness for Longitudinal Data in Acute Kidney Injury with Sepsis. 8](#_Toc133519625)

[Figures. The kmlShape package Generated Longitudinal Plots for Each Cluster 9](#_Toc133519626)

[Fig S2. For pH 9](#_Toc133519627)

[Fig S3. For LD 9](#_Toc133519628)

[Fig S4. For Creatinine 10](#_Toc133519629)

[Fig S5. For Lactate 10](#_Toc133519630)

[Fig S6. For Platelet Values 11](#_Toc133519631)

[Fig S7. For Bicarbonate 11](#_Toc133519632)

[Fig S8. For Albumin Values 12](#_Toc133519633)

[Fig S9. For Glucose Values 12](#_Toc133519634)

[Fig S10. For BUN 13](#_Toc133519635)

[Figures. The longitudinal Profile base on Actual Data. (A. Blood culture positive group, B. Blood culture negative group) 14](#_Toc133519636)

[Fig S11. For pH 14](#_Toc133519637)

[Fig. S12 For LD 14](#_Toc133519638)

[Fig. S13 for Creatinine 15](#_Toc133519639)

[Fig S14. For lactate 15](#_Toc133519640)

[Fig S15. For platelet 16](#_Toc133519641)

[Fig S16. For Bicarbonate 16](#_Toc133519642)

[Fig S17. For Albumin 17](#_Toc133519643)

[Fig S18. For Glucose 17](#_Toc133519644)

[Fig S19. For BUN 18](#_Toc133519645)

[Tables. The Characteristics and levels Change over time in Positive/Negative Group 19](#_Toc133519646)

[Table S1. For pH Levels 19](#_Toc133519647)

[Table S2. For LD Levels 20](#_Toc133519648)

[Table S3. For Creatinine levels 21](#_Toc133519649)

[Table S4. For Lactate levels 22](#_Toc133519650)

[Table S5. For Platelet Levels 23](#_Toc133519651)

[Table S6. For Bicarbonate Levels 24](#_Toc133519652)

[Table S7. For Albumin levels 25](#_Toc133519653)

[Table S8. For Glucose levels 26](#_Toc133519654)

[Table S9. For BUN levels 27](#_Toc133519655)

# STROBE Statement—Checklist of items that should be included in reports of cohort studies

|  | | | Item No | Recommendation | Checked | |  |
| --- | --- | --- | --- | --- | --- | --- | --- |
| **Title and abstract** | | | 1 | (*a*) Indicate the study’s design with a commonly used term in the title or the abstract | Yes | |  |
|  |  |  |  | (*b*) Provide in the abstract an informative and balanced summary of what was done and what was found | Yes | |  |
| Introduction | | | | | | |  |
| Background/rationale | | | 2 | Explain the scientific background and rationale for the investigation being reported | Yes | |  |
| Objectives | | | 3 | State specific objectives, including any prespecified hypotheses | Yes | |  |
| Methods | | | | | | |  |
| Study design | | | 4 | Present key elements of study design early in the paper | Yes | |  |
| Setting | | | 5 | Describe the setting, locations, and relevant dates, including periods of recruitment, exposure, follow-up, and data collection | Yes | |  |
| Participants | | | 6 | (*a*) Give the eligibility criteria, and the sources and methods of selection of participants. Describe methods of follow-up | Yes | |  |
|  |  |  |  | (*b*) For matched studies, give matching criteria and number of exposed and unexposed | Yes | |  |
| Variables | | | 7 | Clearly define all outcomes, exposures, predictors, potential confounders, and effect modifiers. Give diagnostic criteria, if applicable | Yes | |  |
| Data sources/ measurement | | | 8* | For each variable of interest, give sources of data and details of methods of assessment (measurement). Describe comparability of assessment methods if there is more than one group | Yes | |  |
| Bias | | | 9 | Describe any efforts to address potential sources of bias | Yes | |  |
| Study size | | | 10 | Explain how the study size was arrived at | No | |  |
| Quantitative variables | | | 11 | Explain how quantitative variables were handled in the analyses. If applicable, describe which groupings were chosen and why | Yes | |  |
| Statistical methods | | | 12 | (*a*) Describe all statistical methods, including those used to control for confounding | Yes | |  |
|  |  |  |  | (*b*) Describe any methods used to examine subgroups and interactions | Yes | |  |
|  |  |  |  | (*c*) Explain how missing data were addressed | Yes | |  |
|  |  |  |  | (*d*) If applicable, explain how loss to follow-up was addressed | NA | |  |
|  |  |  |  | (*e*) Describe any sensitivity analyses | NA | |  |
| Results | | | | |  | |  |
| Participants | | | 13* | (a) Report numbers of individuals at each stage of study—eg numbers potentially eligible, examined for eligibility, confirmed eligible, included in the study, completing follow-up, and analysed | Yes | |  |
|  |  |  |  | (b) Give reasons for non-participation at each stage | NA | |  |
|  |  |  |  | (c) Consider use of a flow diagram | Yes | |  |
| Descriptive data | | | 14* | (a) Give characteristics of study participants (eg demographic, clinical, social) and information on exposures and potential confounders | Yes | |  |
|  |  |  |  | (b) Indicate number of participants with missing data for each variable of interest | Yes | |  |
|  |  |  |  | (c) Summarise follow-up time (eg, average and total amount) | NA | |  |
| Outcome data | | | 15* | Report numbers of outcome events or summary measures over time | Yes | |  |
| Main results | 16 | (*a*) Give unadjusted estimates and, if applicable, confounder-adjusted estimates and their precision (eg, 95% confidence interval). Make clear which confounders were adjusted for and why they were included | | | | Yes | |
|  |  | (*b*) Report category boundaries when continuous variables were categorized | | | | Yes | |
|  |  | (*c*) If relevant, consider translating estimates of relative risk into absolute risk for a meaningful time period | | | | Yes | |
| Other analyses | 17 | Report other analyses done—eg analyses of subgroups and interactions, and sensitivity analyses | | | | Yes | |
| Discussion | | | | | | | |
| Key results | 18 | Summarise key results with reference to study objectives | | | | Yes | |
| Limitations | 19 | Discuss limitations of the study, taking into account sources of potential bias or imprecision. Discuss both direction and magnitude of any potential bias | | | | Yes | |
| Interpretation | 20 | Give a cautious overall interpretation of results considering objectives, limitations, multiplicity of analyses, results from similar studies, and other relevant evidence | | | | Yes | |
| Generalisability | 21 | Discuss the generalisability (external validity) of the study results | | | | Yes | |
| Other information | | | | | | | |
| Funding | 22 | Give the source of funding and the role of the funders for the present study and, if applicable, for the original study on which the present article is based | | | | Yes | |

*Give information separately for exposed and unexposed groups.

**Note:** An Explanation and Elaboration article discusses each checklist item and gives methodological background and published examples of transparent reporting. The STROBE checklist is best used in conjunction with this article (freely available on the Web sites of PLoS Medicine at http://www.plosmedicine.org/, Annals of Internal Medicine at http://www.annals.org/, and Epidemiology at http://www.epidem.com/). Information on the STROBE Initiative is available at http://www.strobe-statement.org.

## Figure S1. Plot of Missingness for Longitudinal Data in Acute Kidney Injury with Sepsis.


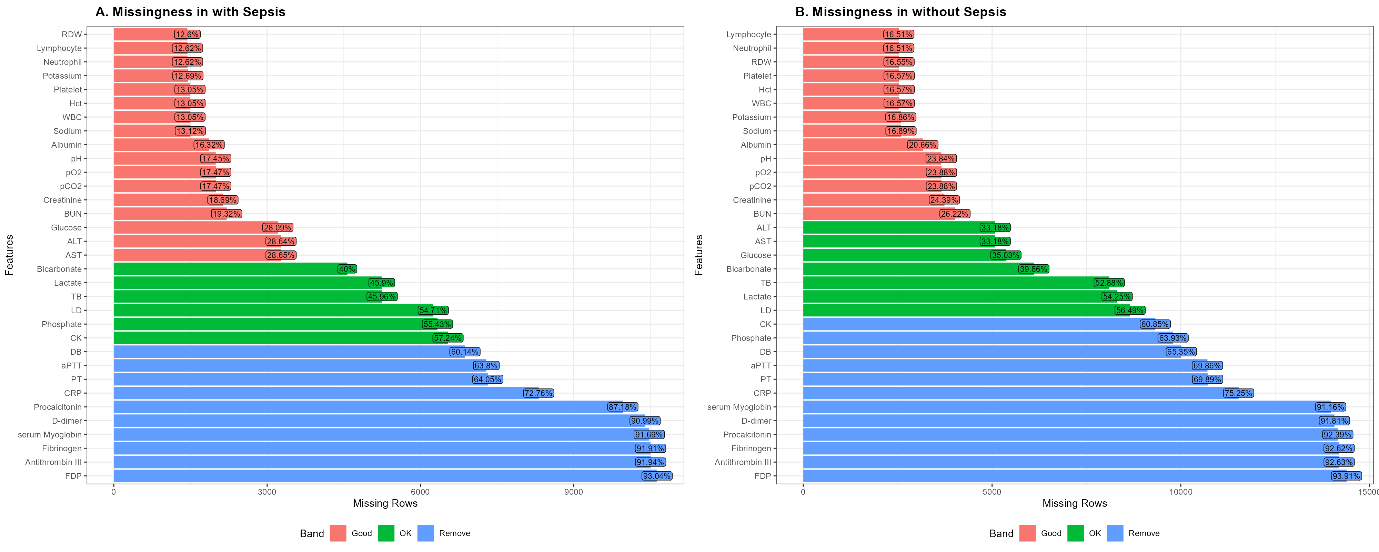


# Figures. The kmlShape package Generated Longitudinal Plots for Each Cluster

## Fig S2. For pH


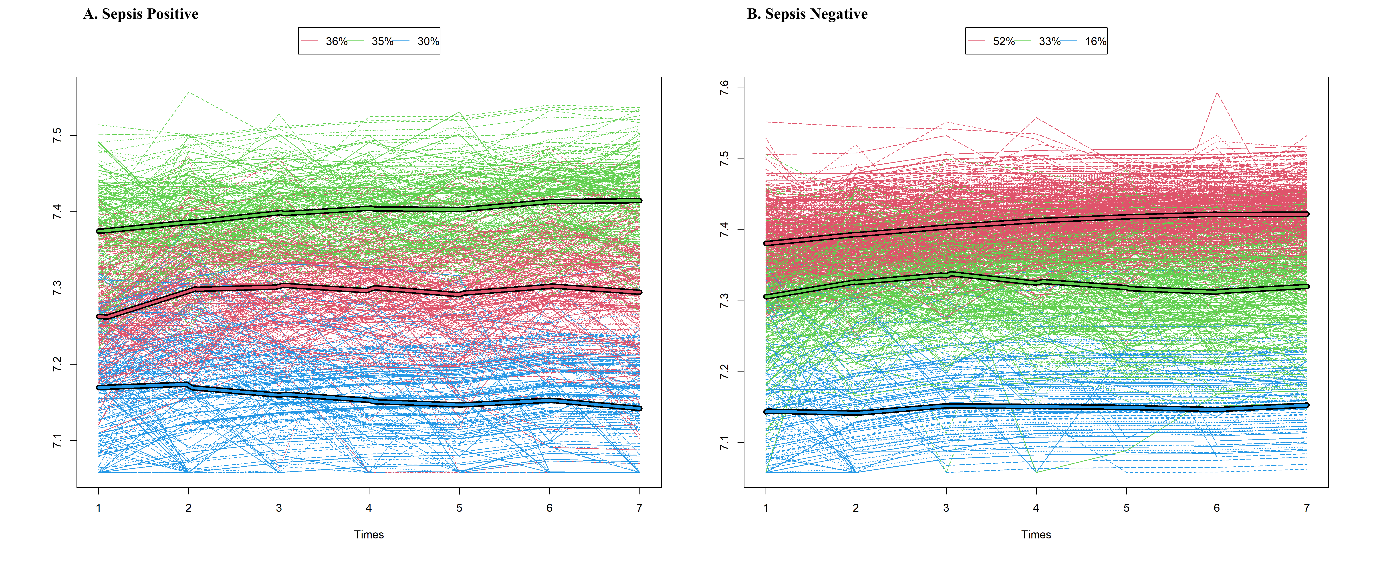


## Fig S3. For LD


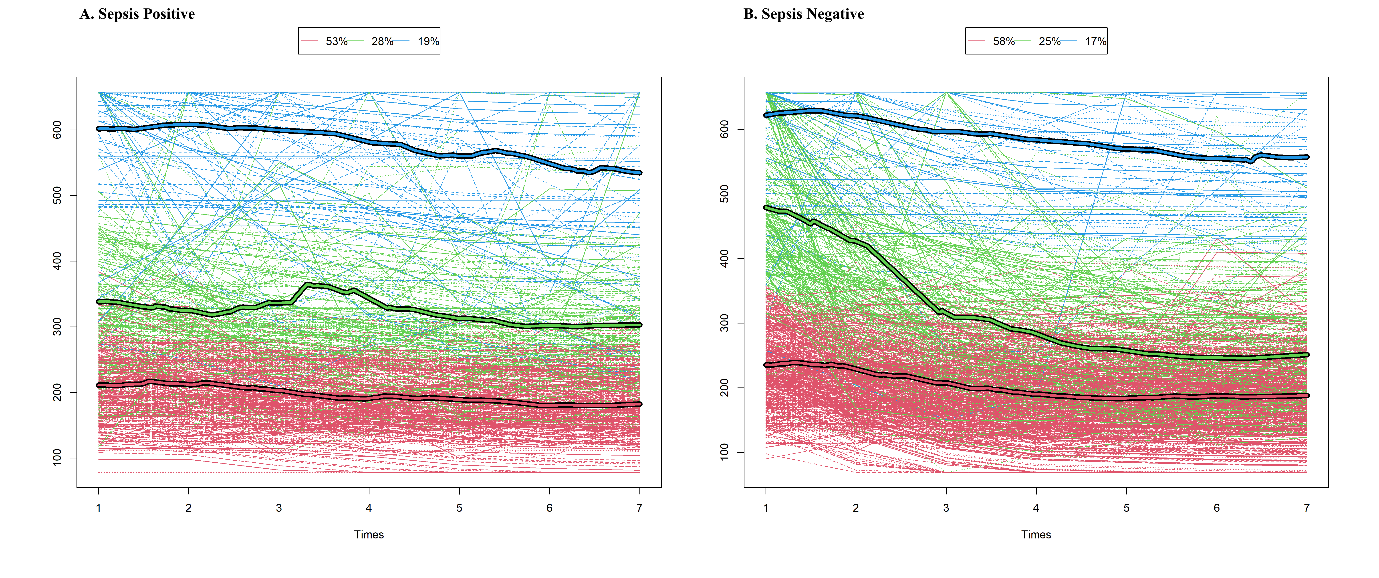


## Fig S4. For Creatinine


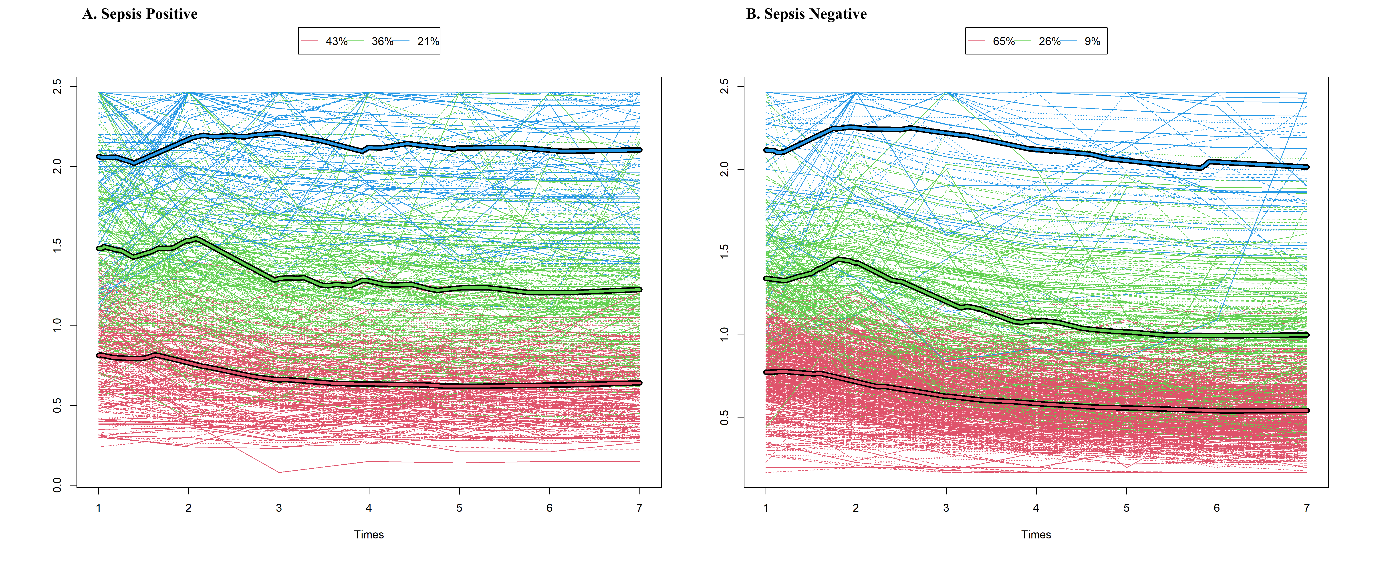


## Fig S5. For Lactate


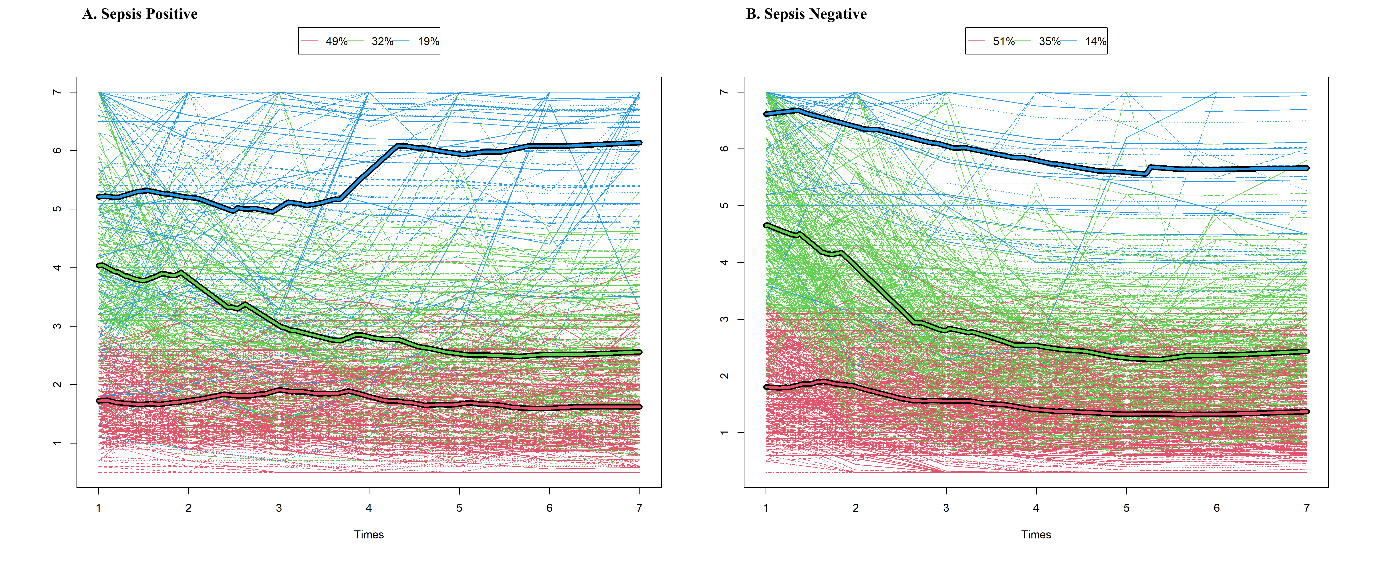


## Fig S6. For Platelet Values


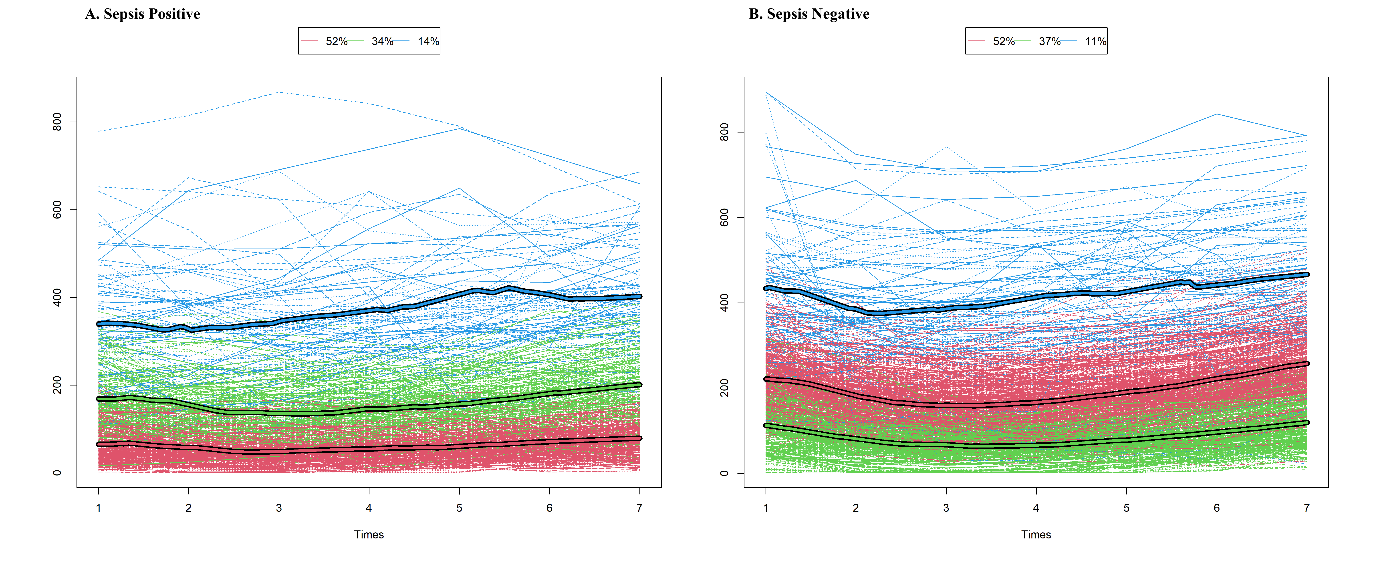


## Fig S7. For Bicarbonate


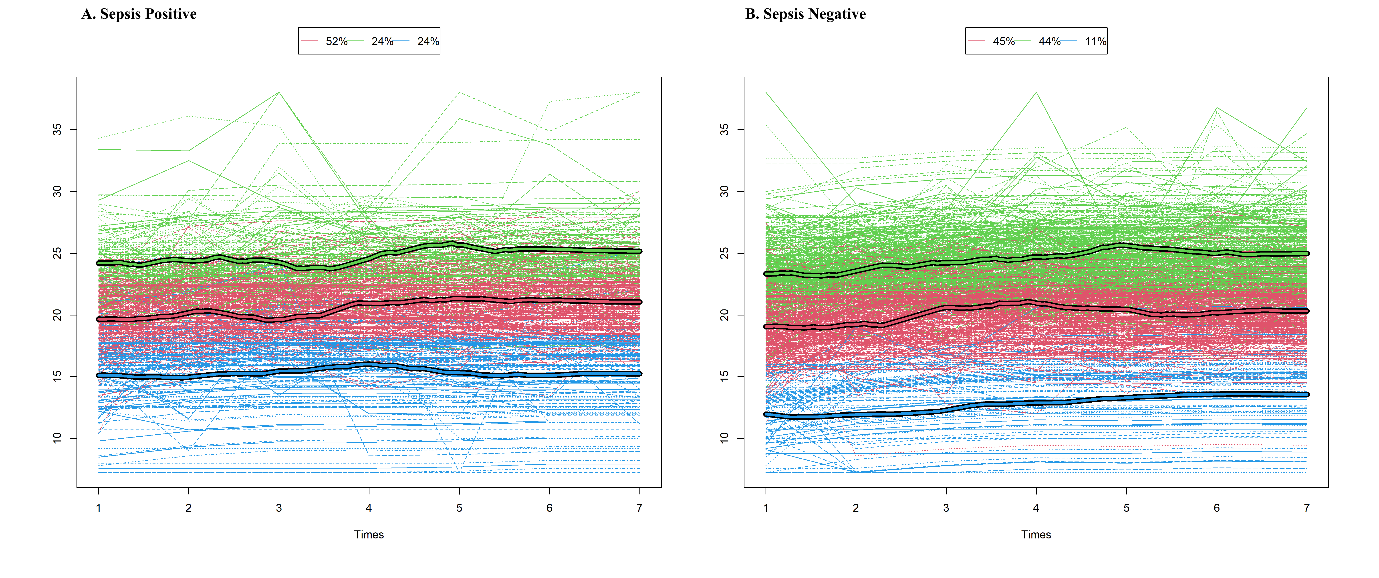


## Fig S8. For Albumin Values


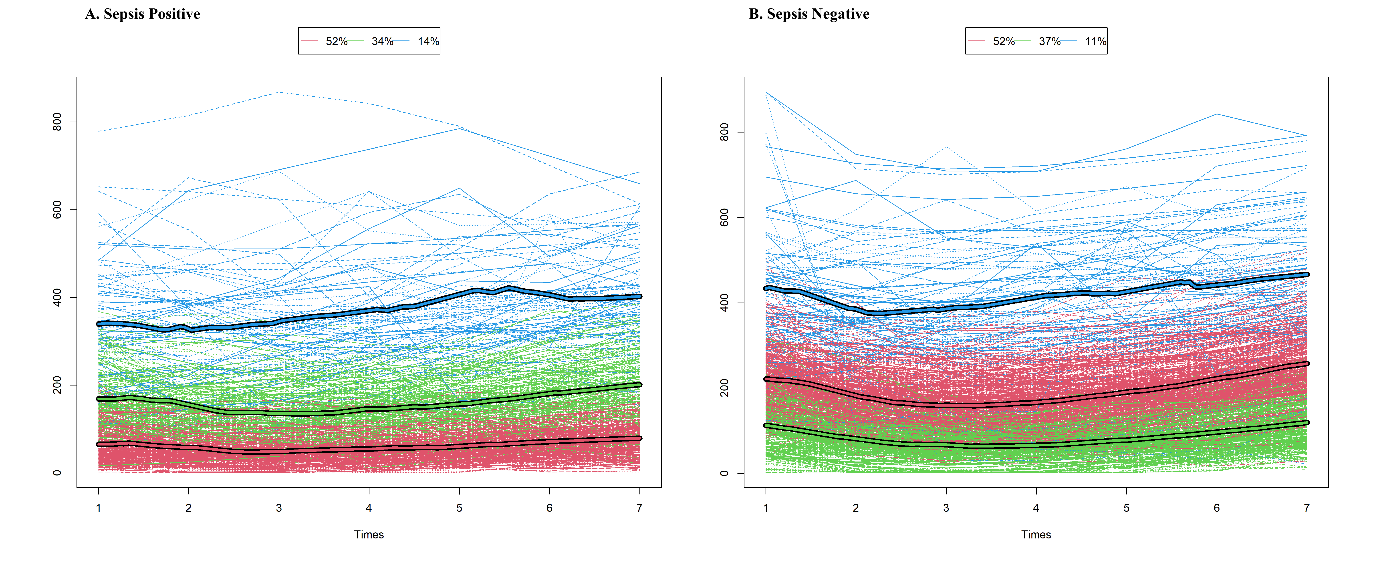


## Fig S9. For Glucose Values


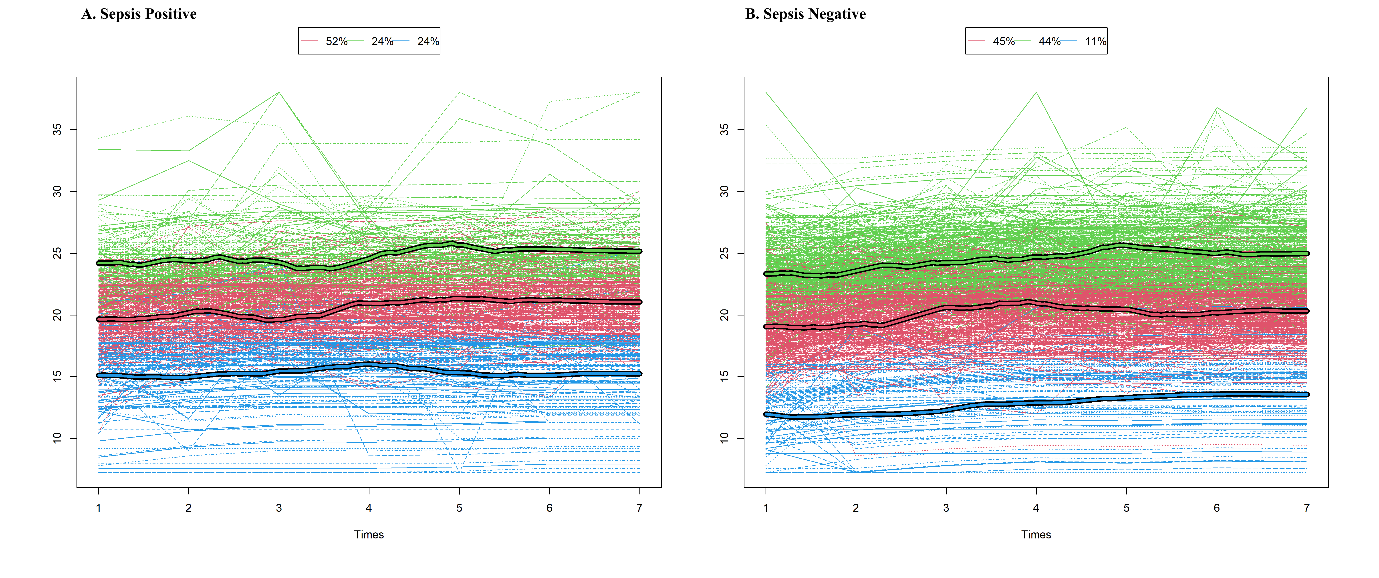


## Fig S10. For BUN


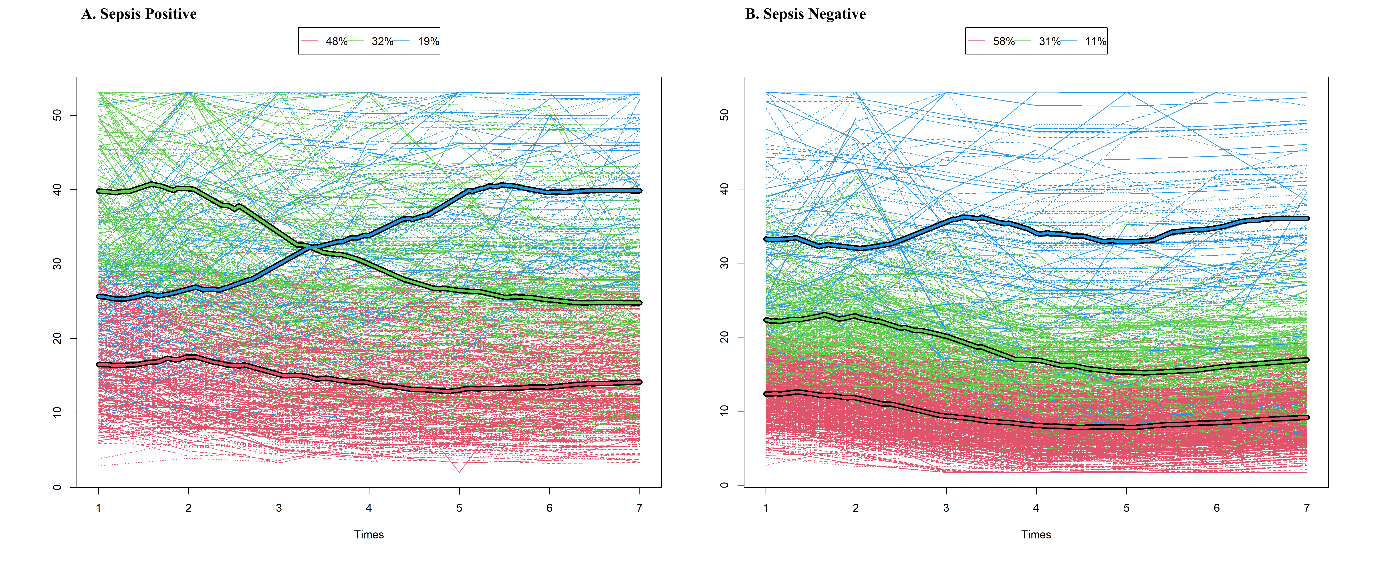


# Figures. The Raincloud Plot base on Actual Data. (A. Blood culture positive group, B. Blood culture negative group)

## Fig S11. For pH


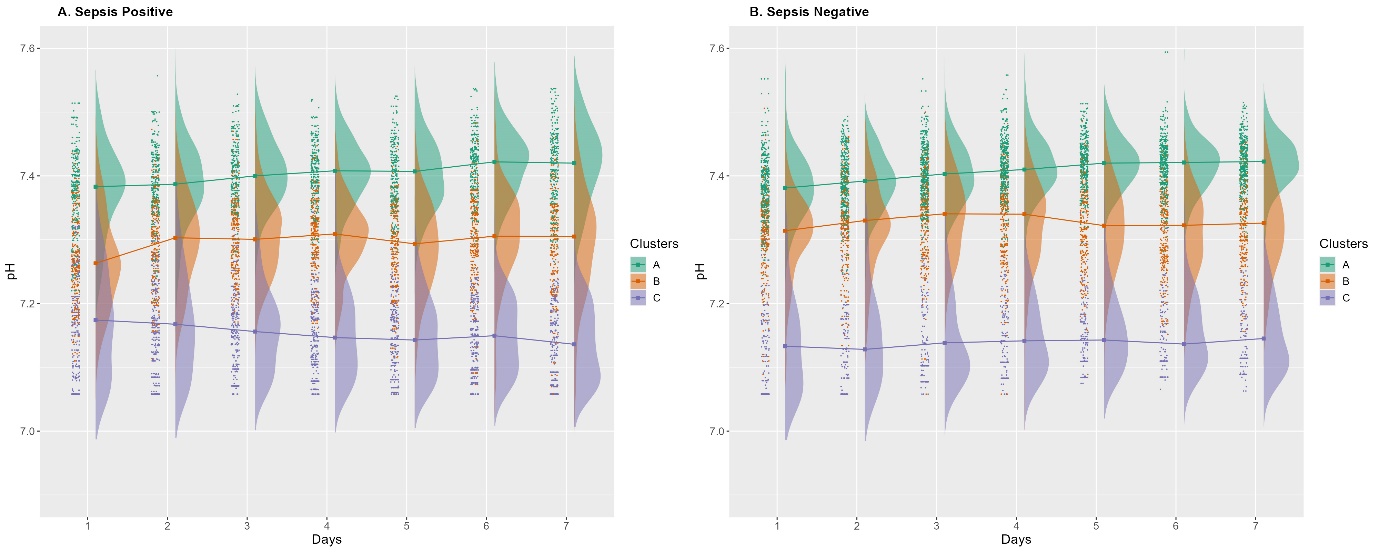


## Fig. S12 For LD


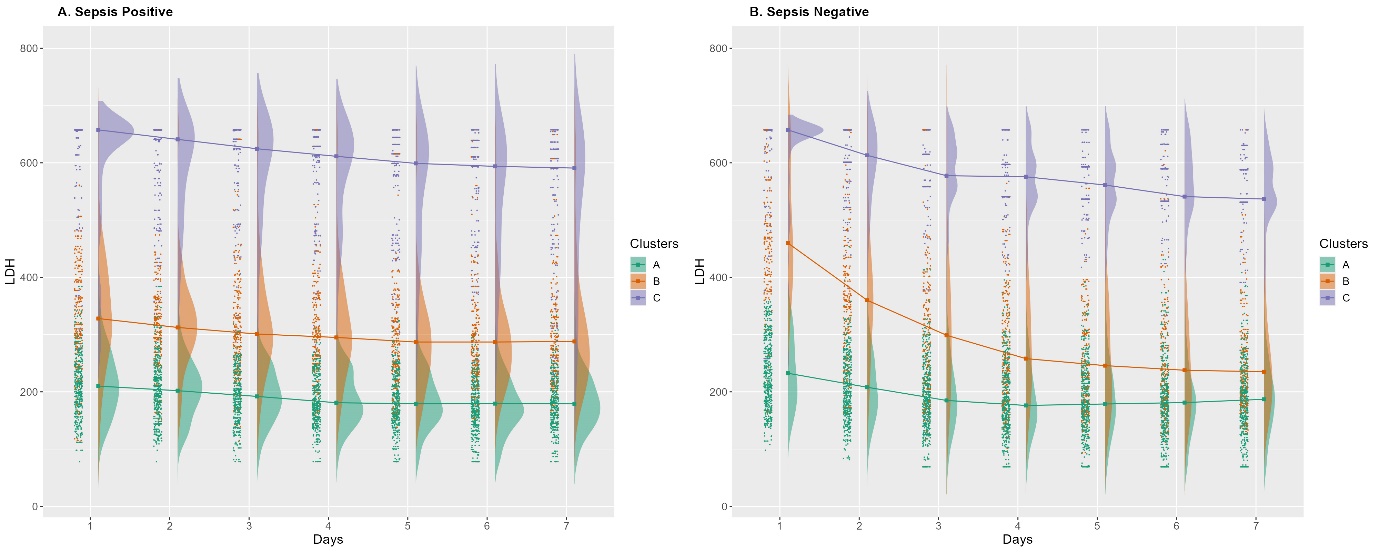


## Fig. S13 for Creatinine


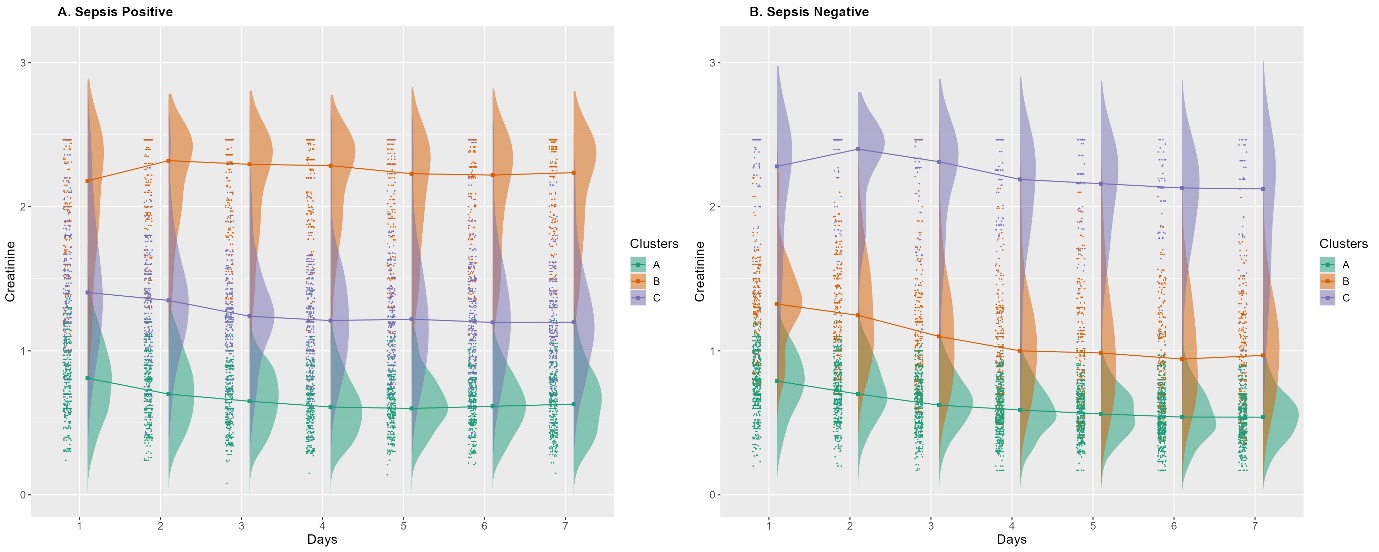


## Fig S14. For platelet


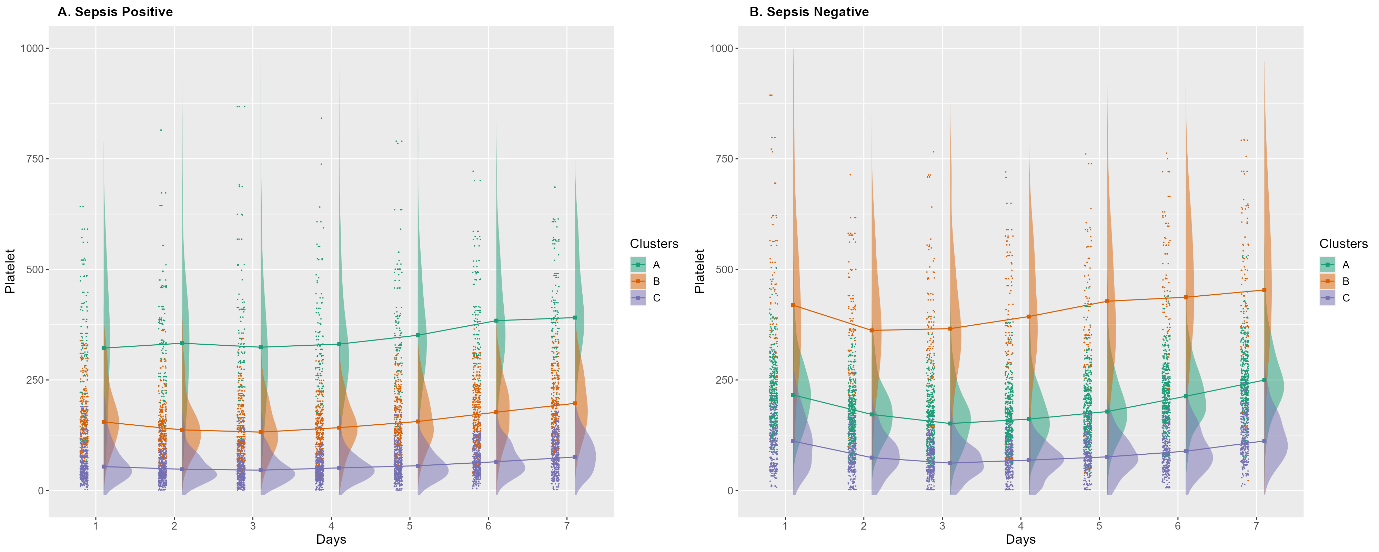


## Fig S15. For lactate


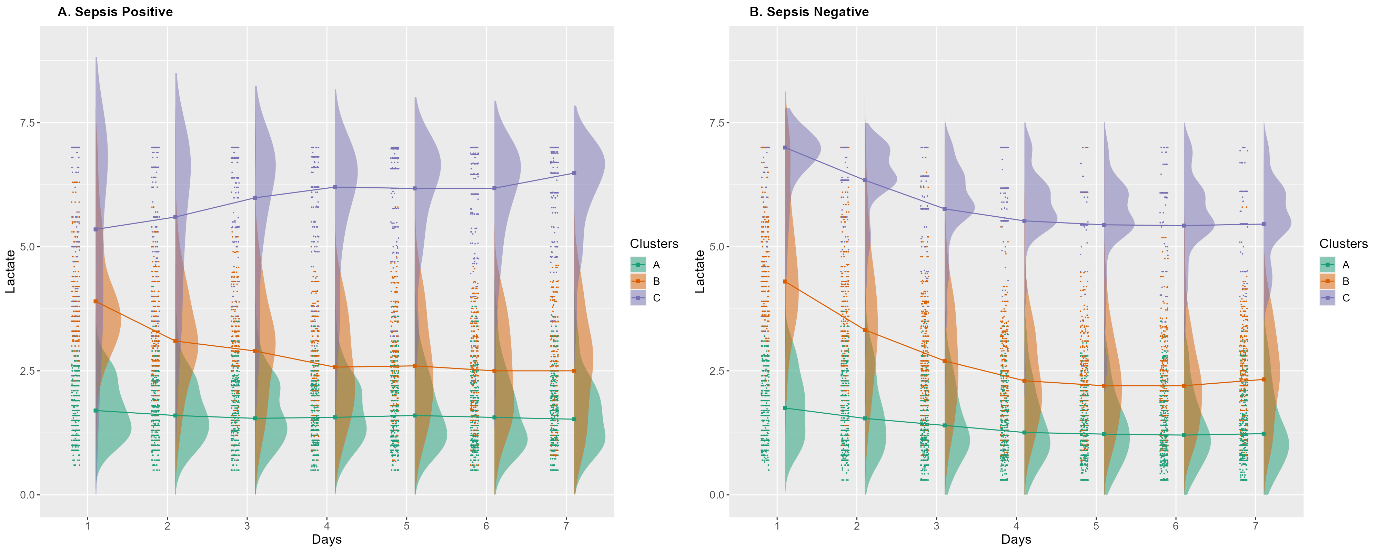


## Fig S16. For Bicarbonate


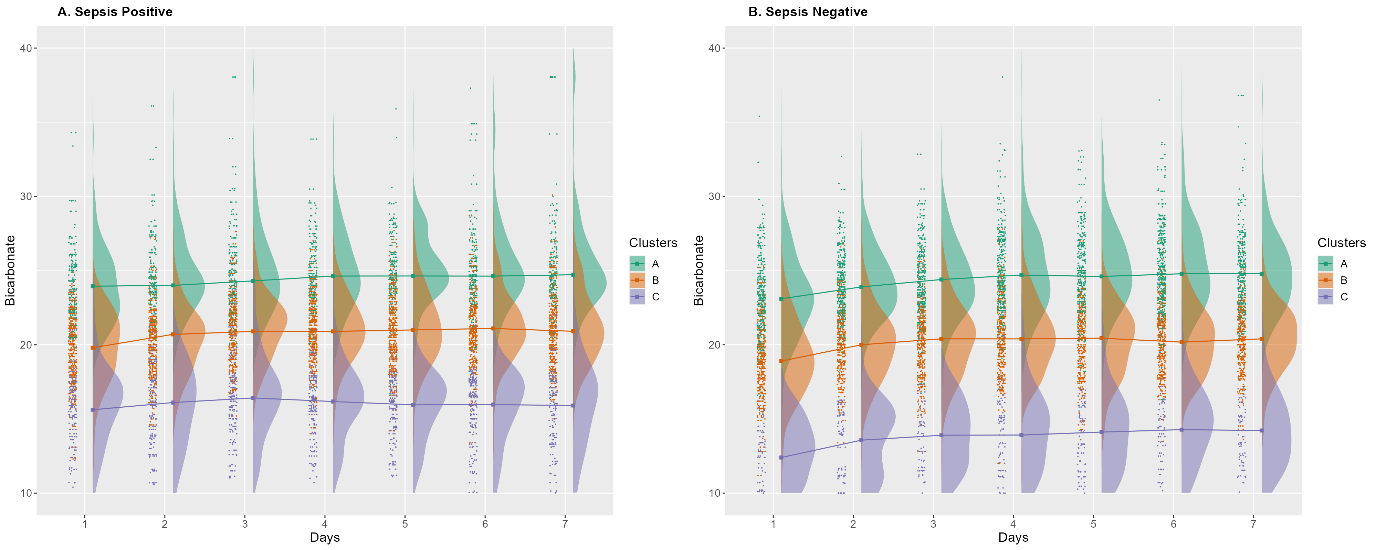


## Fig S17. For Albumin


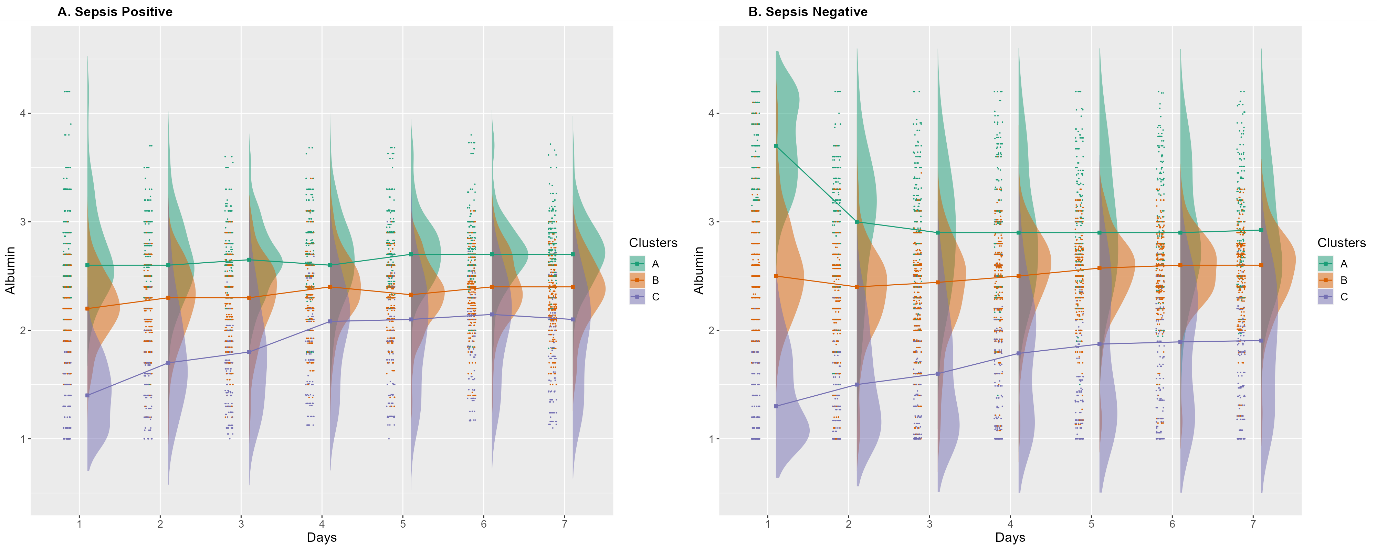


## Fig S18. For Glucose


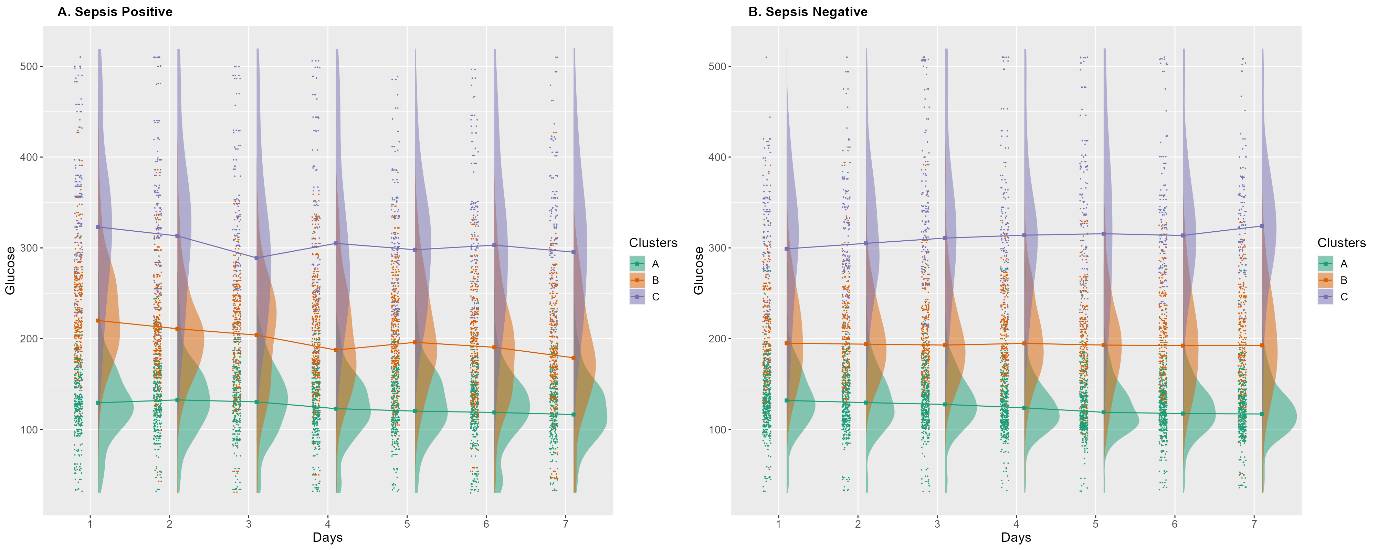


## Fig S19. For BUN


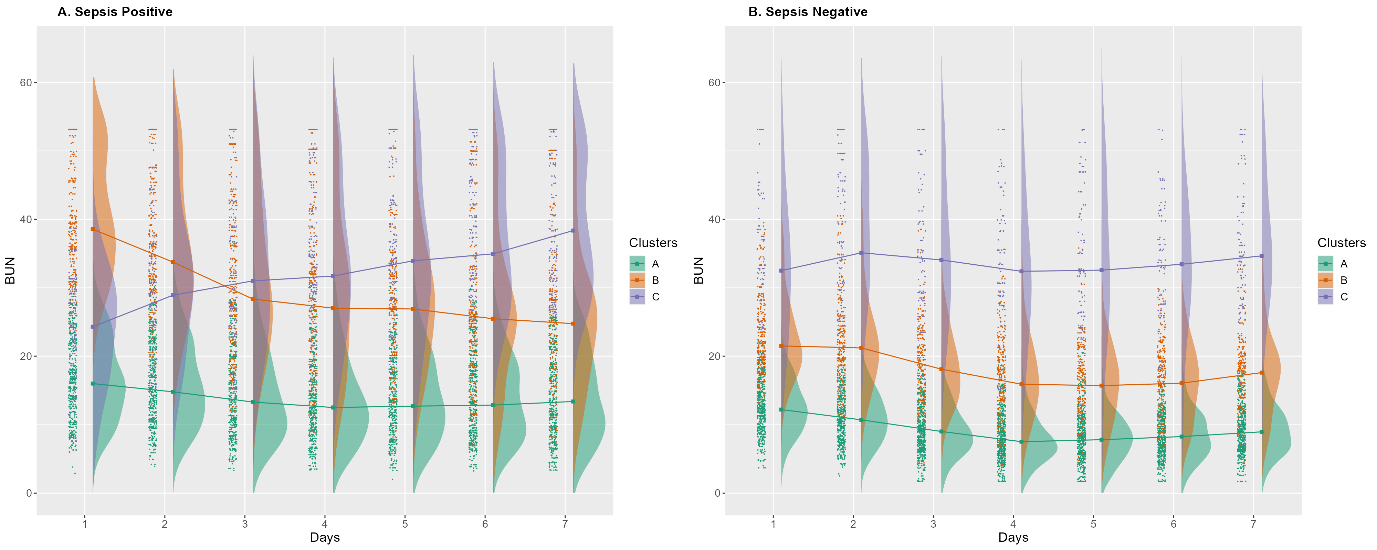


# Tables. The Characteristics and levels Change over time in Positive/Negative Group

## Table S1. For pH Levels

|  |  | Sepsis Positive | | | | Sepsis Negative | | | |
| --- | --- | --- | --- | --- | --- | --- | --- | --- | --- |
| Group | Variables | A, N = 186 (34.6%) | B, N = 192 (35.8%) | C, N = 159 (29.6%) | p-value | A, N = 420 (51.4%) | B, N = 268 (32.8%) | C, N = 129 (15.8%) | p-value |
| Demographics | Mortality | 33 (17.7%) | 115 (59.9%) | 156 (98.1%) | <0.001 | 25 (6.0%) | 117 (43.7%) | 126 (97.7%) | <0.001 |
|  | Patient Age |  |  |  | 0.575 |  |  |  | <0.001 |
|  | Median [IQR] | 54 [44, 66] | 53 [43, 64] | 54 [46, 64] |  | 49 [40, 59] | 51 [41, 62] | 56 [45, 71] |  |
|  | Sex |  |  |  | 0.513 |  |  |  | 0.094 |
|  | Male | 158 (84.9%) | 162 (84.4%) | 128 (80.5%) |  | 331 (78.8%) | 226 (84.3%) | 99 (76.7%) |  |
|  | Female | 28 (15.1%) | 30 (15.6%) | 31 (19.5%) |  | 89 (21.2%) | 42 (15.7%) | 30 (23.3%) |  |
|  | TBSA |  |  |  | <0.001 |  |  |  | <0.001 |
|  | Median [IQR] | 30 [20, 45] | 52 [32, 70] | 63 [46, 83] |  | 24 [15, 34] | 46 [26, 70] | 75 [47, 88] |  |
|  | Inhalation | 73 (39.2%) | 111 (57.8%) | 97 (61.0%) | <0.001 | 138 (32.9%) | 146 (54.5%) | 102 (79.1%) | <0.001 |
|  | LOICU |  |  |  | <0.001 |  |  |  | <0.001 |
|  | Median [IQR] | 29 [19, 46] | 27 [17, 42] | 11 [6, 17] |  | 17 [6, 31] | 23 [10, 38] | 3 [2, 8] |  |
| pH value | Day 1 |  |  |  | <0.001 |  |  |  | <0.001 |
|  | Median [IQR] | 7.38 [7.33, 7.42] | 7.26 [7.22, 7.31] | 7.17 [7.10, 7.22] |  | 7.38 [7.35, 7.41] | 7.31 [7.26, 7.35] | 7.13 [7.06, 7.20] |  |
|  | Day 2 |  |  |  | <0.001 |  |  |  | <0.001 |
|  | Median [IQR] | 7.39 [7.35, 7.43] | 7.30 [7.24, 7.34] | 7.17 [7.11, 7.23] |  | 7.39 [7.36, 7.43] | 7.33 [7.29, 7.37] | 7.13 [7.08, 7.18] |  |
|  | Day 3 |  |  |  | <0.001 |  |  |  | <0.001 |
|  | Median [IQR] | 7.40 [7.37, 7.44] | 7.30 [7.26, 7.34] | 7.16 [7.10, 7.20] |  | 7.40 [7.37, 7.43] | 7.34 [7.29, 7.37] | 7.14 [7.10, 7.20] |  |
|  | Day 4 |  |  |  | <0.001 |  |  |  | <0.001 |
|  | Median [IQR] | 7.41 [7.37, 7.44] | 7.31 [7.26, 7.34] | 7.15 [7.10, 7.19] |  | 7.41 [7.38, 7.44] | 7.34 [7.28, 7.37] | 7.14 [7.11, 7.19] |  |
|  | Day 5 |  |  |  | <0.001 |  |  |  | <0.001 |
|  | Median [IQR] | 7.41 [7.36, 7.44] | 7.29 [7.24, 7.34] | 7.14 [7.10, 7.19] |  | 7.42 [7.39, 7.44] | 7.32 [7.28, 7.37] | 7.14 [7.11, 7.19] |  |
|  | Day 6 |  |  |  | <0.001 |  |  |  | <0.001 |
|  | Median [IQR] | 7.42 [7.38, 7.45] | 7.31 [7.27, 7.35] | 7.15 [7.10, 7.20] |  | 7.42 [7.39, 7.45] | 7.32 [7.27, 7.36] | 7.14 [7.11, 7.19] |  |
|  | Day 7 |  |  |  | <0.001 |  |  |  | <0.001 |
|  | Median [IQR] | 7.42 [7.37, 7.45] | 7.30 [7.25, 7.35] | 7.14 [7.09, 7.19] |  | 7.42 [7.40, 7.45] | 7.33 [7.27, 7.36] | 7.15 [7.11, 7.19] |  |
| Mean of overall median value | pH |  |  |  | <0.001 |  |  |  | <0.001 |
|  | Median [IQR] | 7.40 [7.36, 7.43] | 7.29 [7.24, 7.34] | 7.18 [7.12, 7.22] |  | 7.41 [7.38, 7.44] | 7.33 [7.28, 7.36] | 7.17 [7.11, 7.22] |  |

## Table S2. For LD Levels

|  |  | Sepsis Positive | | | | Sepsis Negative | | | |
| --- | --- | --- | --- | --- | --- | --- | --- | --- | --- |
| Group | Variables | A, N = 285 (53.0%) | B, N = 153 (28.4%) | C, N = 100 (18.6%) | p-value | A, N = 473 (57.7%) | B, N = 205 (25.0%) | C, N = 142 (17.3%) | p-value |
| Demographics | Mortality | 146 (51.2%) | 80 (52.3%) | 79 (79.0%) | <0.001 | 71 (15.0%) | 79 (38.5%) | 116 (81.7%) | <0.001 |
|  | Patient Age |  |  |  | 0.075 |  |  |  | 0.528 |
|  | Median [IQR] | 55 [46, 65] | 53 [41, 66] | 51 [41, 60] |  | 51 [41, 61] | 51 [39, 63] | 53 [42, 67] |  |
|  | Sex |  |  |  | 0.221 |  |  |  | 0.382 |
|  | Male | 245 (86.0%) | 122 (79.7%) | 82 (82.0%) |  | 384 (81.2%) | 166 (81.0%) | 108 (76.1%) |  |
|  | Female | 40 (14.0%) | 31 (20.3%) | 18 (18.0%) |  | 89 (18.8%) | 39 (19.0%) | 34 (23.9%) |  |
|  | TBSA |  |  |  | 0.227 |  |  |  | <0.001 |
|  | Median [IQR] | 45 [28, 67] | 43 [29, 63] | 58 [31, 73] |  | 26 [17, 42] | 40 [25, 61] | 69 [33, 90] |  |
|  | Inhalation | 149 (52.3%) | 70 (45.8%) | 62 (62.0%) | 0.039 | 189 (40.0%) | 92 (44.9%) | 103 (72.5%) | <0.001 |
|  | LOICU |  |  |  | <0.001 |  |  |  | <0.001 |
|  | Median [IQR] | 25 [14, 41] | 24 [13, 38] | 10 [4, 23] |  | 18 [7, 32] | 22 [9, 38] | 3 [2, 7] |  |
| LDH value | Day 1 |  |  |  | <0.001 |  |  |  | <0.001 |
|  | Median [IQR] | 210 [174, 250] | 328 [276, 403] | 658 [570, 658] |  | 232 [190, 282] | 460 [402, 570] | 658 [656, 658] |  |
|  | Day 2 |  |  |  | <0.001 |  |  |  | <0.001 |
|  | Median [IQR] | 202 [169, 238] | 312 [269, 363] | 641 [514, 658] |  | 208 [169, 247] | 360 [285, 434] | 613 [601, 658] |  |
|  | Day 3 |  |  |  | <0.001 |  |  |  | <0.001 |
|  | Median [IQR] | 192 [162, 231] | 301 [260, 364] | 625 [508, 658] |  | 185 [152, 229] | 299 [214, 380] | 577 [558, 615] |  |
|  | Day 4 |  |  |  | <0.001 |  |  |  | <0.001 |
|  | Median [IQR] | 181 [156, 222] | 295 [243, 352] | 611 [482, 645] |  | 176 [141, 223] | 258 [196, 343] | 576 [541, 597] |  |
|  | Day 5 |  |  |  | <0.001 |  |  |  | <0.001 |
|  | Median [IQR] | 179 [153, 214] | 287 [235, 338] | 599 [459, 632] |  | 179 [142, 221] | 246 [185, 325] | 561 [537, 593] |  |
|  | Day 6 |  |  |  | <0.001 |  |  |  | <0.001 |
|  | Median [IQR] | 179 [151, 213] | 287 [239, 341] | 594 [450, 627] |  | 181 [144, 224] | 238 [182, 311] | 541 [533, 589] |  |
|  | Day 7 |  |  |  | <0.001 |  |  |  | <0.001 |
|  | Median [IQR] | 179 [150, 216] | 288 [230, 341] | 591 [451, 624] |  | 187 [148, 227] | 235 [187, 300] | 537 [531, 588] |  |
| Mean of overall median value | LDH |  |  |  | <0.001 |  |  |  | <0.001 |
|  | Median [IQR] | 188 [160, 225] | 295 [245, 349] | 592 [461, 658] |  | 202 [162, 245] | 286 [206, 385] | 593 [531, 658] |  |

## Table S3. For Creatinine levels

|  |  | Sepsis Positive | | | | Sepsis Negative | | | |
| --- | --- | --- | --- | --- | --- | --- | --- | --- | --- |
| Group | Variables | A, N = 229 (42.6%) | B, N = 113 (21.0%) | C, N = 196 (36.4%) | p-value | A, N = 535 (65.2%) | B, N = 212 (25.9%) | C, N = 73 (8.90%) | p-value |
| Demographics | Mortality | 79 (34.5%) | 79 (69.9%) | 147 (75.0%) | <0.001 | 87 (16.3%) | 119 (56.1%) | 60 (82.2%) | <0.001 |
|  | Patient Age |  |  |  | 0.120 |  |  |  | <0.001 |
|  | Median [IQR] | 53 [43, 62] | 52 [43, 61] | 55 [47, 66] |  | 49 [39, 59] | 56 [47, 68] | 51 [41, 72] |  |
|  | Sex |  |  |  | <0.001 |  |  |  | 0.002 |
|  | Male | 175 (76.4%) | 102 (90.3%) | 172 (87.8%) |  | 411 (76.8%) | 182 (85.8%) | 65 (89.0%) |  |
|  | Female | 54 (23.6%) | 11 (9.7%) | 24 (12.2%) |  | 124 (23.2%) | 30 (14.2%) | 8 (11.0%) |  |
|  | TBSA |  |  |  | <0.001 |  |  |  | <0.001 |
|  | Median [IQR] | 40 [24, 60] | 52 [37, 70] | 56 [33, 75] |  | 28 [17, 45] | 45 [25, 76] | 61 [30, 88] |  |
|  | Inhalation | 116 (50.7%) | 70 (61.9%) | 95 (48.5%) | 0.061 | 223 (41.7%) | 116 (54.7%) | 46 (63.0%) | <0.001 |
|  | LOICU |  |  |  | <0.001 |  |  |  | <0.001 |
|  | Median [IQR] | 25 [16, 40] | 14 [8, 35] | 19 [10, 34] |  | 17 [6, 34] | 13 [5, 30] | 5 [3, 13] |  |
| Creatinine value | Day 1 |  |  |  | <0.001 |  |  |  | <0.001 |
|  | Median [IQR] | 0.81 [0.60, 1.00] | 2.18 [1.77, 2.46] | 1.41 [1.17, 1.79] |  | 0.79 [0.64, 0.91] | 1.33 [1.18, 1.50] | 2.28 [1.72, 2.46] |  |
|  | Day 2 |  |  |  | <0.001 |  |  |  | <0.001 |
|  | Median [IQR] | 0.70 [0.57, 0.88] | 2.32 [1.95, 2.46] | 1.35 [1.10, 1.54] |  | 0.70 [0.58, 0.86] | 1.25 [1.00, 1.50] | 2.40 [2.08, 2.46] |  |
|  | Day 3 |  |  |  | <0.001 |  |  |  | <0.001 |
|  | Median [IQR] | 0.65 [0.50, 0.80] | 2.29 [1.89, 2.46] | 1.24 [1.04, 1.46] |  | 0.62 [0.50, 0.75] | 1.10 [0.84, 1.38] | 2.31 [1.95, 2.46] |  |
|  | Day 4 |  |  |  | <0.001 |  |  |  | <0.001 |
|  | Median [IQR] | 0.61 [0.48, 0.77] | 2.29 [1.88, 2.46] | 1.21 [1.00, 1.47] |  | 0.59 [0.48, 0.70] | 1.00 [0.75, 1.27] | 2.19 [1.87, 2.38] |  |
|  | Day 5 |  |  |  | <0.001 |  |  |  | <0.001 |
|  | Median [IQR] | 0.60 [0.48, 0.76] | 2.23 [1.86, 2.45] | 1.22 [0.96, 1.44] |  | 0.56 [0.45, 0.68] | 0.99 [0.70, 1.24] | 2.16 [1.85, 2.36] |  |
|  | Day 6 |  |  |  | <0.001 |  |  |  | <0.001 |
|  | Median [IQR] | 0.61 [0.49, 0.78] | 2.22 [1.83, 2.42] | 1.20 [0.96, 1.42] |  | 0.54 [0.43, 0.66] | 0.94 [0.70, 1.21] | 2.13 [1.82, 2.33] |  |
|  | Day 7 |  |  |  | <0.001 |  |  |  | <0.001 |
|  | Median [IQR] | 0.63 [0.47, 0.80] | 2.24 [1.86, 2.42] | 1.20 [0.98, 1.45] |  | 0.54 [0.42, 0.66] | 0.97 [0.73, 1.20] | 2.12 [1.81, 2.32] |  |
| Mean of overall median value | Creatinine |  |  |  | <0.001 |  |  |  | <0.001 |
|  | Median [IQR] | 0.64 [0.48, 0.80] | 2.29 [1.84, 2.46] | 1.21 [0.99, 1.51] |  | 0.60 [0.50, 0.74] | 1.00 [0.77, 1.31] | 2.25 [1.83, 2.46] |  |

## Table S4. For Platelet Levels

|  |  | Sepsis Positive | | | | Sepsis Negative | | | |
| --- | --- | --- | --- | --- | --- | --- | --- | --- | --- |
| Group | Variables | A, N = 77 (14.3%) | B, N = 181 (33.6%) | C, N = 280 (52.0%) | p-value | A, N = 431 (52.4%) | B, N = 88 (10.7%) | C, N = 303 (36.9%) | p-value |
| Demographics | Mortality | 12 (15.6%) | 64 (35.4%) | 229 (81.8%) | <0.001 | 68 (15.8%) | 20 (22.7%) | 179 (59.1%) | <0.001 |
|  | Patient Age |  |  |  | 0.744 |  |  |  | 0.055 |
|  | Median [IQR] | 54 [45, 62] | 54 [45, 65] | 54 [42, 64] |  | 49 [40, 61] | 52 [41, 61] | 53 [43, 64] |  |
|  | Sex |  |  |  | 0.784 |  |  |  | 0.891 |
|  | Male | 64 (83.1%) | 154 (85.1%) | 231 (82.5%) |  | 343 (79.6%) | 72 (81.8%) | 245 (80.9%) |  |
|  | Female | 13 (16.9%) | 27 (14.9%) | 49 (17.5%) |  | 88 (20.4%) | 16 (18.2%) | 58 (19.1%) |  |
|  | TBSA |  |  |  | <0.001 |  |  |  | <0.001 |
|  | Median [IQR] | 33 [16, 45] | 37 [22, 55] | 60 [40, 80] |  | 25 [16, 40] | 32 [17, 52] | 51 [30, 78] |  |
|  | Inhalation | 31 (40.3%) | 91 (50.3%) | 159 (56.8%) | 0.032 | 160 (37.1%) | 44 (50.0%) | 181 (59.7%) | <0.001 |
|  | LOICU |  |  |  | <0.001 |  |  |  | 0.100 |
|  | Median [IQR] | 30 [20, 42] | 28 [17, 44] | 15 [10, 27] |  | 13 [5, 28] | 25 [6, 35] | 13 [5, 32] |  |
| Platelet value | Day 1 |  |  |  | <0.001 |  |  |  | <0.001 |
|  | Median [IQR] | 322 [222, 425] | 155 [115, 212] | 54 [35, 90] |  | 216 [166, 262] | 419 [328, 507] | 111 [62, 147] |  |
|  | Day 2 |  |  |  | <0.001 |  |  |  | <0.001 |
|  | Median [IQR] | 333 [234, 393] | 137 [104, 180] | 48 [31, 72] |  | 172 [132, 213] | 362 [292, 484] | 74 [48, 104] |  |
|  | Day 3 |  |  |  | <0.001 |  |  |  | <0.001 |
|  | Median [IQR] | 324 [248, 409] | 132 [101, 178] | 46 [28, 67] |  | 151 [116, 200] | 366 [303, 478] | 62 [41, 89] |  |
|  | Day 4 |  |  |  | <0.001 |  |  |  | <0.001 |
|  | Median [IQR] | 331 [273, 424] | 142 [106, 187] | 51 [32, 69] |  | 161 [123, 211] | 393 [315, 488] | 69 [44, 91] |  |
|  | Day 5 |  |  |  | <0.001 |  |  |  | <0.001 |
|  | Median [IQR] | 351 [291, 436] | 156 [122, 201] | 56 [37, 79] |  | 178 [143, 228] | 428 [333, 518] | 76 [54, 105] |  |
|  | Day 6 |  |  |  | <0.001 |  |  |  | <0.001 |
|  | Median [IQR] | 384 [321, 477] | 177 [136, 222] | 65 [45, 95] |  | 213 [176, 258] | 437 [356, 540] | 89 [63, 129] |  |
|  | Day 7 |  |  |  | <0.001 |  |  |  | <0.001 |
|  | Median [IQR] | 391 [322, 486] | 197 [150, 245] | 76 [50, 105] |  | 250 [211, 301] | 453 [372, 560] | 112 [76, 156] |  |
| Mean of overall median value | Platelet |  |  |  | <0.001 |  |  |  | <0.001 |
|  | Median [IQR] | 341 [270, 425] | 155 [114, 209] | 57 [37, 90] |  | 189 [141, 242] | 415 [325, 509] | 83 [58, 118] |  |

## Table S5. For Lactate levels

|  |  | Sepsis Positive | | | | Sepsis Negative | | | |
| --- | --- | --- | --- | --- | --- | --- | --- | --- | --- |
| Group | Variables | A, N = 263 (48.9%) | B, N = 173 (32.2%) | C, N = 102 (19.0%) | p-value | A, N = 409 (50.6%) | B, N = 283 (35.0%) | C, N = 117 (14.5%) | p-value |
| Demographics | Mortality | 102 (38.8%) | 106 (61.3%) | 97 (95.1%) | <0.001 | 55 (13.4%) | 100 (35.3%) | 111 (94.9%) | <0.001 |
|  | Patient Age |  |  |  | 0.008 |  |  |  | <0.001 |
|  | Median [IQR] | 56 [47, 66] | 51 [38, 62] | 54 [46, 63] |  | 51 [41, 62] | 49 [39, 60] | 56 [47, 72] |  |
|  | Sex |  |  |  | 0.053 |  |  |  | 0.128 |
|  | Male | 226 (85.9%) | 146 (84.4%) | 77 (75.5%) |  | 331 (80.9%) | 233 (82.3%) | 86 (73.5%) |  |
|  | Female | 37 (14.1%) | 27 (15.6%) | 25 (24.5%) |  | 78 (19.1%) | 50 (17.7%) | 31 (26.5%) |  |
|  | TBSA |  |  |  | <0.001 |  |  |  | <0.001 |
|  | Median [IQR] | 39 [22, 60] | 55 [31, 70] | 60 [43, 80] |  | 24 [15, 38] | 40 [26, 62] | 76 [50, 92] |  |
|  | Inhalation | 126 (47.9%) | 103 (59.5%) | 52 (51.0%) | 0.055 | 148 (36.2%) | 138 (48.8%) | 97 (82.9%) | <0.001 |
|  | LOICU |  |  |  | <0.001 |  |  |  | <0.001 |
|  | Median [IQR] | 27 [18, 43] | 22 [12, 38] | 10 [5, 18] |  | 18 [7, 31] | 21 [9, 36] | 2 [2, 5] |  |
| Lactate value | Day 1 |  |  |  | <0.001 |  |  |  | <0.001 |
|  | Median [IQR] | 1.70 [1.30, 2.20] | 3.90 [3.20, 4.70] | 5.35 [3.60, 7.00] |  | 1.75 [1.20, 2.40] | 4.30 [3.68, 5.50] | 7.00 [6.60, 7.00] |  |
|  | Day 2 |  |  |  | <0.001 |  |  |  | <0.001 |
|  | Median [IQR] | 1.60 [1.20, 2.20] | 3.10 [2.43, 3.90] | 5.60 [4.03, 6.60] |  | 1.54 [1.10, 2.20] | 3.33 [2.60, 4.30] | 6.34 [6.34, 7.00] |  |
|  | Day 3 |  |  |  | <0.001 |  |  |  | <0.001 |
|  | Median [IQR] | 1.54 [1.20, 2.20] | 2.90 [2.08, 3.50] | 5.99 [3.83, 6.79] |  | 1.40 [0.92, 1.90] | 2.70 [2.00, 3.46] | 5.76 [5.76, 6.42] |  |
|  | Day 4 |  |  |  | <0.001 |  |  |  | <0.001 |
|  | Median [IQR] | 1.56 [1.10, 2.10] | 2.58 [1.80, 3.40] | 6.20 [4.90, 6.68] |  | 1.26 [0.87, 1.88] | 2.30 [1.70, 3.01] | 5.52 [5.52, 6.18] |  |
|  | Day 5 |  |  |  | <0.001 |  |  |  | <0.001 |
|  | Median [IQR] | 1.60 [1.07, 2.15] | 2.60 [1.82, 3.50] | 6.18 [5.05, 6.79] |  | 1.23 [0.80, 1.72] | 2.20 [1.60, 2.91] | 5.44 [5.44, 6.10] |  |
|  | Day 6 |  |  |  | <0.001 |  |  |  | <0.001 |
|  | Median [IQR] | 1.56 [1.10, 2.07] | 2.50 [1.70, 3.33] | 6.18 [5.27, 6.67] |  | 1.21 [0.80, 1.80] | 2.20 [1.50, 2.91] | 5.43 [5.43, 6.09] |  |
|  | Day 7 |  |  |  | <0.001 |  |  |  | <0.001 |
|  | Median [IQR] | 1.53 [1.10, 2.10] | 2.50 [1.70, 3.30] | 6.48 [5.84, 6.86] |  | 1.23 [0.82, 1.88] | 2.33 [1.70, 3.02] | 5.46 [5.46, 6.12] |  |
| Mean of overall median value | Lactate |  |  |  | <0.001 |  |  |  | <0.001 |
|  | Median [IQR] | 1.66 [1.20, 2.19] | 2.80 [1.90, 3.60] | 5.50 [3.90, 6.70] |  | 1.47 [1.01, 2.00] | 2.60 [1.90, 3.60] | 5.92 [5.10, 6.76] |  |

## Table S6. For Bicarbonate Levels

|  |  | Sepsis Positive | | | | Sepsis Negative | | | |
| --- | --- | --- | --- | --- | --- | --- | --- | --- | --- |
| Group | Variables | A, N = 128 (24.4%) | B, N = 270 (51.5%) | C, N = 126 (24.0%) | p-value | A, N = 356 (44.0%) | B, N = 366 (45.2%) | C, N = 87 (10.8%) | p-value |
| Demographics | Mortality | 50 (39.1%) | 151 (55.9%) | 95 (75.4%) | <0.001 | 44 (12.4%) | 142 (38.8%) | 78 (89.7%) | <0.001 |
|  | Patient Age |  |  |  | 0.168 |  |  |  | <0.001 |
|  | Median [IQR] | 54 [43, 65] | 52 [42, 63] | 56 [48, 64] |  | 49 [39, 58] | 51 [42, 64] | 57 [46, 70] |  |
|  | Sex |  |  |  | 0.537 |  |  |  | 0.027 |
|  | Male | 111 (86.7%) | 222 (82.2%) | 105 (83.3%) |  | 300 (84.3%) | 287 (78.4%) | 64 (73.6%) |  |
|  | Female | 17 (13.3%) | 48 (17.8%) | 21 (16.7%) |  | 56 (15.7%) | 79 (21.6%) | 23 (26.4%) |  |
|  | TBSA |  |  |  | <0.001 |  |  |  | <0.001 |
|  | Median [IQR] | 38 [22, 60] | 50 [30, 65] | 56 [33, 77] |  | 24 [15, 40] | 40 [24, 63] | 70 [36, 86] |  |
|  | Inhalation | 68 (53.1%) | 147 (54.4%) | 61 (48.4%) | 0.542 | 143 (40.2%) | 173 (47.3%) | 67 (77.0%) | <0.001 |
|  | LOICU |  |  |  | <0.001 |  |  |  | <0.001 |
|  | Median [IQR] | 24 [13, 37] | 25 [13, 40] | 14 [6, 27] |  | 15 [6, 31] | 19 [8, 35] | 2 [2, 6] |  |
| Bicarbonate value | Day 1 |  |  |  | <0.001 |  |  |  | <0.001 |
|  | Median [IQR] | 24.0 [22.4, 25.5] | 19.8 [18.1, 21.4] | 15.6 [13.4, 17.1] |  | 23.1 [21.7, 24.8] | 18.9 [17.4, 20.6] | 12.4 [10.1, 14.1] |  |
|  | Day 2 |  |  |  | <0.001 |  |  |  | <0.001 |
|  | Median [IQR] | 24.0 [23.0, 26.0] | 20.7 [19.0, 22.0] | 16.1 [13.7, 17.6] |  | 23.9 [22.6, 25.5] | 20.0 [18.3, 21.5] | 13.6 [11.0, 14.9] |  |
|  | Day 3 |  |  |  | <0.001 |  |  |  | <0.001 |
|  | Median [IQR] | 24.3 [23.0, 26.2] | 20.9 [19.4, 22.2] | 16.4 [14.2, 17.7] |  | 24.4 [22.7, 26.0] | 20.4 [18.6, 21.6] | 13.9 [11.4, 15.6] |  |
|  | Day 4 |  |  |  | <0.001 |  |  |  | <0.001 |
|  | Median [IQR] | 24.6 [23.4, 26.6] | 20.9 [19.4, 22.4] | 16.2 [14.1, 17.5] |  | 24.7 [22.9, 26.4] | 20.4 [18.8, 21.8] | 13.9 [11.5, 15.7] |  |
|  | Day 5 |  |  |  | <0.001 |  |  |  | <0.001 |
|  | Median [IQR] | 24.6 [23.5, 26.1] | 21.0 [19.5, 22.4] | 15.9 [13.6, 17.5] |  | 24.6 [22.8, 26.6] | 20.4 [18.7, 21.8] | 14.1 [11.5, 15.7] |  |
|  | Day 6 |  |  |  | <0.001 |  |  |  | <0.001 |
|  | Median [IQR] | 24.6 [23.5, 26.4] | 21.1 [19.7, 22.5] | 16.0 [13.6, 17.6] |  | 24.8 [23.0, 26.8] | 20.2 [18.7, 21.7] | 14.3 [11.6, 15.7] |  |
|  | Day 7 |  |  |  | <0.001 |  |  |  | <0.001 |
|  | Median [IQR] | 24.7 [23.5, 26.5] | 20.9 [19.5, 22.4] | 15.9 [13.4, 17.4] |  | 24.8 [23.0, 26.5] | 20.4 [19.0, 21.8] | 14.2 [11.5, 15.7] |  |
| Mean of overall median value | Bicarbonate |  |  |  | <0.001 |  |  |  | <0.001 |
|  | Median [IQR] | 24.4 [23.1, 26.1] | 20.9 [19.2, 22.3] | 16.3 [13.8, 17.7] |  | 24.0 [22.3, 26.0] | 20.2 [18.3, 21.7] | 14.5 [11.7, 16.0] |  |

## Table S7. For Albumin levels

|  |  | Sepsis Positive | | | | Sepsis Negative | | | |
| --- | --- | --- | --- | --- | --- | --- | --- | --- | --- |
| Group | Variables | A, N = 177 (32.9%) | B, N = 269 (50.0%) | C, N = 92 (17.1%) | p-value | A, N = 191 (23.3%) | B, N = 460 (56.1%) | C, N = 169 (20.6%) | p-value |
| Demographics | Mortality | 61 (34.5%) | 165 (61.3%) | 79 (85.9%) | <0.001 | 11 (5.8%) | 115 (25.0%) | 139 (82.2%) | <0.001 |
|  | Patient Age |  |  |  | 0.004 |  |  |  | 0.082 |
|  | Median [IQR] | 55 [44, 68] | 55 [46, 64] | 50 [38, 59] |  | 48 [41, 56] | 52 [41, 63] | 52 [41, 64] |  |
|  | Sex |  |  |  | 0.797 |  |  |  | 0.611 |
|  | Male | 145 (81.9%) | 226 (84.0%) | 78 (84.8%) |  | 158 (82.7%) | 365 (79.3%) | 135 (79.9%) |  |
|  | Female | 32 (18.1%) | 43 (16.0%) | 14 (15.2%) |  | 33 (17.3%) | 95 (20.7%) | 34 (20.1%) |  |
|  | TBSA |  |  |  | <0.001 |  |  |  | <0.001 |
|  | Median [IQR] | 33 [16, 50] | 53 [33, 70] | 63 [47, 83] |  | 19 [10, 25] | 32 [21, 50] | 70 [47, 85] |  |
|  | Inhalation | 81 (45.8%) | 140 (52.0%) | 60 (65.2%) | 0.009 | 60 (31.4%) | 201 (43.7%) | 122 (72.2%) | <0.001 |
|  | LOICU |  |  |  | <0.001 |  |  |  | <0.001 |
|  | Median [IQR] | 25 [13, 37] | 23 [13, 41] | 12 [5, 26] |  | 8 [4, 23] | 22 [9, 36] | 6 [2, 19] |  |
| Albumin value | Day 1 |  |  |  | <0.001 |  |  |  | <0.001 |
|  | Median [IQR] | 2.60 [2.50, 2.90] | 2.20 [2.00, 2.40] | 1.40 [1.10, 1.60] |  | 3.70 [3.40, 4.10] | 2.50 [2.30, 2.80] | 1.30 [1.03, 1.70] |  |
|  | Day 2 |  |  |  | <0.001 |  |  |  | <0.001 |
|  | Median [IQR] | 2.60 [2.40, 2.90] | 2.30 [2.10, 2.50] | 1.70 [1.39, 2.03] |  | 3.00 [2.60, 3.37] | 2.40 [2.10, 2.60] | 1.50 [1.00, 1.90] |  |
|  | Day 3 |  |  |  | <0.001 |  |  |  | <0.001 |
|  | Median [IQR] | 2.65 [2.40, 2.82] | 2.30 [2.10, 2.50] | 1.80 [1.40, 2.10] |  | 2.90 [2.50, 3.30] | 2.44 [2.14, 2.64] | 1.60 [1.04, 2.10] |  |
|  | Day 4 |  |  |  | <0.001 |  |  |  | <0.001 |
|  | Median [IQR] | 2.60 [2.40, 2.83] | 2.40 [2.20, 2.60] | 2.08 [1.60, 2.30] |  | 2.90 [2.50, 3.32] | 2.50 [2.28, 2.73] | 1.79 [1.14, 2.20] |  |
|  | Day 5 |  |  |  | <0.001 |  |  |  | <0.001 |
|  | Median [IQR] | 2.70 [2.50, 2.90] | 2.33 [2.20, 2.50] | 2.10 [1.71, 2.40] |  | 2.90 [2.60, 3.35] | 2.57 [2.30, 2.74] | 1.87 [1.17, 2.40] |  |
|  | Day 6 |  |  |  | <0.001 |  |  |  | <0.001 |
|  | Median [IQR] | 2.70 [2.50, 2.87] | 2.40 [2.20, 2.60] | 2.15 [1.79, 2.42] |  | 2.90 [2.60, 3.45] | 2.60 [2.36, 2.76] | 1.89 [1.26, 2.30] |  |
|  | Day 7 |  |  |  | <0.001 |  |  |  | <0.001 |
|  | Median [IQR] | 2.70 [2.60, 2.90] | 2.40 [2.20, 2.60] | 2.10 [1.76, 2.40] |  | 2.92 [2.60, 3.41] | 2.60 [2.38, 2.78] | 1.91 [1.28, 2.32] |  |
| Mean of overall median value | Albumin |  |  |  | <0.001 |  |  |  | <0.001 |
|  | Median [IQR] | 2.63 [2.43, 2.85] | 2.40 [2.20, 2.50] | 2.10 [1.60, 2.30] |  | 2.80 [2.50, 3.40] | 2.50 [2.21, 2.70] | 1.97 [1.40, 2.30] |  |

## Table S8. For Glucose levels

|  |  | Sepsis Positive | | | | Sepsis Negative | | | |
| --- | --- | --- | --- | --- | --- | --- | --- | --- | --- |
| Group | Variables | A, N = 212 (39.8%) | B, N = 219 (41.2%) | C, N = 101 (19.0%) | p-value | A, N = 420 (52.0%) | B, N = 253 (31.3%) | C, N = 135 (16.7%) | p-value |
| Demographics | Mortality | 101 (47.6%) | 131 (59.8%) | 70 (69.3%) | 0.002 | 65 (15.5%) | 111 (43.9%) | 88 (65.2%) | <0.001 |
|  | Patient Age |  |  |  | 0.039 |  |  |  | <0.001 |
|  | Median [IQR] | 53 [40, 63] | 54 [46, 64] | 56 [48, 68] |  | 48 [38, 58] | 53 [44, 65] | 56 [47, 71] |  |
|  | Sex |  |  |  | 0.201 |  |  |  | 0.437 |
|  | Male | 183 (86.3%) | 182 (83.1%) | 79 (78.2%) |  | 344 (81.9%) | 198 (78.3%) | 106 (78.5%) |  |
|  | Female | 29 (13.7%) | 37 (16.9%) | 22 (21.8%) |  | 76 (18.1%) | 55 (21.7%) | 29 (21.5%) |  |
|  | TBSA |  |  |  | 0.004 |  |  |  | <0.001 |
|  | Median [IQR] | 41 [25, 61] | 50 [32, 70] | 58 [32, 74] |  | 26 [16, 40] | 40 [23, 72] | 52 [26, 80] |  |
|  | Inhalation | 101 (47.6%) | 123 (56.2%) | 57 (56.4%) | 0.151 | 157 (37.4%) | 140 (55.3%) | 84 (62.2%) | <0.001 |
|  | LOICU |  |  |  | 0.235 |  |  |  | <0.001 |
|  | Median [IQR] | 23 [12, 36] | 21 [13, 40] | 19 [10, 35] |  | 13 [5, 28] | 19 [8, 36] | 12 [4, 32] |  |
| Glucose value | Day 1 |  |  |  | <0.001 |  |  |  | <0.001 |
|  | Median [IQR] | 130 [106, 156] | 220 [190, 265] | 323 [273, 373] |  | 132 [112, 154] | 195 [164, 235] | 299 [238, 340] |  |
|  | Day 2 |  |  |  | <0.001 |  |  |  | <0.001 |
|  | Median [IQR] | 133 [110, 157] | 211 [175, 246] | 313 [253, 362] |  | 130 [111, 153] | 194 [168, 227] | 305 [272, 362] |  |
|  | Day 3 |  |  |  | <0.001 |  |  |  | <0.001 |
|  | Median [IQR] | 130 [107, 153] | 204 [160, 242] | 289 [243, 348] |  | 128 [109, 148] | 193 [163, 225] | 311 [266, 361] |  |
|  | Day 4 |  |  |  | <0.001 |  |  |  | <0.001 |
|  | Median [IQR] | 123 [101, 147] | 188 [154, 232] | 305 [252, 354] |  | 124 [106, 145] | 195 [167, 226] | 314 [269, 358] |  |
|  | Day 5 |  |  |  | <0.001 |  |  |  | <0.001 |
|  | Median [IQR] | 120 [101, 146] | 196 [162, 242] | 298 [240, 346] |  | 119 [104, 141] | 193 [164, 229] | 316 [263, 367] |  |
|  | Day 6 |  |  |  | <0.001 |  |  |  | <0.001 |
|  | Median [IQR] | 119 [99, 143] | 191 [150, 229] | 303 [252, 349] |  | 118 [101, 140] | 192 [163, 227] | 314 [273, 360] |  |
|  | Day 7 |  |  |  | <0.001 |  |  |  | <0.001 |
|  | Median [IQR] | 116 [96, 145] | 179 [144, 219] | 295 [244, 342] |  | 117 [101, 137] | 193 [162, 232] | 324 [275, 368] |  |
| Mean of overall median value | Glucose |  |  |  | <0.001 |  |  |  | <0.001 |
|  | Median [IQR] | 130 [106, 152] | 202 [164, 245] | 290 [233, 342] |  | 128 [110, 150] | 193 [163, 226] | 301 [244, 357] |  |

## Table S9. For BUN levels

|  |  | Sepsis Positive | | | | Sepsis Negative | | | |
| --- | --- | --- | --- | --- | --- | --- | --- | --- | --- |
| Group | Variables | A, N = 260 (48.3%) | B, N = 174 (32.3%) | C, N = 104 (19.3%) | p-value | A, N = 472 (57.6%) | B, N = 255 (31.1%) | C, N = 92 (11.2%) | p-value |
| Demographics | Mortality | 107 (41.2%) | 123 (70.7%) | 75 (72.1%) | <0.001 | 91 (19.3%) | 107 (42.0%) | 68 (73.9%) | <0.001 |
|  | Patient Age |  |  |  | 0.042 |  |  |  | <0.001 |
|  | Median [IQR] | 52 [42, 62] | 55 [48, 66] | 56 [44, 68] |  | 46 [38, 56] | 56 [47, 68] | 62 [50, 77] |  |
|  | Sex |  |  |  | 0.094 |  |  |  | 0.709 |
|  | Male | 209 (80.4%) | 147 (84.5%) | 93 (89.4%) |  | 375 (79.4%) | 209 (82.0%) | 73 (79.3%) |  |
|  | Female | 51 (19.6%) | 27 (15.5%) | 11 (10.6%) |  | 97 (20.6%) | 46 (18.0%) | 19 (20.7%) |  |
|  | TBSA |  |  |  | <0.001 |  |  |  | <0.001 |
|  | Median [IQR] | 40 [25, 61] | 56 [32, 75] | 54 [33, 71] |  | 28 [17, 43] | 42 [25, 72] | 50 [24, 80] |  |
|  | Inhalation | 141 (54.2%) | 82 (47.1%) | 58 (55.8%) | 0.247 | 215 (45.6%) | 120 (47.1%) | 49 (53.3%) | 0.404 |
|  | LOICU |  |  |  | 0.548 |  |  |  | <0.001 |
|  | Median [IQR] | 22 [11, 39] | 23 [12, 36] | 19 [11, 35] |  | 12 [5, 27] | 24 [6, 40] | 13 [6, 25] |  |
| BUN value | Day 1 |  |  |  | <0.001 |  |  |  | <0.001 |
|  | Median [IQR] | 16 [12, 21] | 39 [33, 48] | 24 [19, 29] |  | 12 [10, 15] | 22 [18, 25] | 32 [25, 42] |  |
|  | Day 2 |  |  |  | <0.001 |  |  |  | <0.001 |
|  | Median [IQR] | 15 [10, 20] | 34 [29, 43] | 29 [21, 36] |  | 11 [8, 14] | 21 [18, 25] | 35 [30, 44] |  |
|  | Day 3 |  |  |  | <0.001 |  |  |  | <0.001 |
|  | Median [IQR] | 13 [10, 19] | 28 [23, 38] | 31 [24, 39] |  | 9 [7, 11] | 18 [14, 22] | 34 [28, 41] |  |
|  | Day 4 |  |  |  | <0.001 |  |  |  | <0.001 |
|  | Median [IQR] | 13 [9, 18] | 27 [20, 35] | 32 [24, 43] |  | 8 [6, 10] | 16 [12, 20] | 32 [26, 40] |  |
|  | Day 5 |  |  |  | <0.001 |  |  |  | <0.001 |
|  | Median [IQR] | 13 [9, 18] | 27 [20, 35] | 34 [27, 46] |  | 8 [6, 10] | 16 [12, 19] | 33 [26, 41] |  |
|  | Day 6 |  |  |  | <0.001 |  |  |  | <0.001 |
|  | Median [IQR] | 13 [9, 17] | 25 [19, 31] | 35 [29, 47] |  | 8 [6, 11] | 16 [12, 20] | 33 [28, 42] |  |
|  | Day 7 |  |  |  | <0.001 |  |  |  | <0.001 |
|  | Median [IQR] | 13 [10, 18] | 25 [18, 30] | 38 [30, 48] |  | 9 [7, 12] | 18 [13, 20] | 35 [29, 42] |  |
| Mean of overall median value | BUN |  |  |  | <0.001 |  |  |  | <0.001 |
|  | Median [IQR] | 14 [10, 19] | 28 [21, 38] | 31 [24, 41] |  | 9 [7, 12] | 18 [14, 22] | 33 [26, 42] |  |
